# Accumulation Patterns of Metabolites Responsible for the Functional Quality of Virgin Olive Oil during Olive Fruit Ontogeny

**DOI:** 10.3390/antiox13010012

**Published:** 2023-12-20

**Authors:** Pilar Luaces, Jesús Expósito, Paula Benabal, Mar Pascual, Carlos Sanz, Ana G. Pérez

**Affiliations:** Department of Biochemistry and Molecular Biology of Plant Products, Instituto de la Grasa, Spanish National Research Council (CSIC), 41013 Seville, Spain; pluaces@ig.csic.es (P.L.); jexposito@ig.csic.es (J.E.); pbenabal@ig.csic.es (P.B.); marpa@ig.csic.es (M.P.); agracia@ig.csic.es (A.G.P.)

**Keywords:** functional quality, *Olea europaea* L., olive fruit, phenolic compounds, tocopherols, virgin olive oil

## Abstract

The health-promoting antioxidant properties of virgin olive oil (VOO) are today considered priority targets in the new olive breeding programs. Given that these properties depend mainly on its phenolic fraction, whose origin lies in the phenolic compounds present in olive fruit, the objective of this study was to provide further insight into the accumulation dynamics of the main antioxidant compounds, including both polar phenolics and lipophilic tocopherols, during the ontogeny of the olive fruit. Data obtained show that, albeit with significant differences, all the studied genotypes share just after fruit set an intense increase in the synthesis of tyrosol and hydroxytyrosol derivatives, by far the main phenolic compounds of the olive fruit, and a subsequent steady decrease along fruit development and ripening. The accumulation dynamics of flavonoids and tocopherols were different from those of tyrosol and hydroxytyrosol derivatives, presenting a peak of synthesis just before the onset of fruit ripening, and then in general, their content decreases throughout the ripening phase. In the case of flavonoids, all genotypes also share a strong increase in the accumulation of anthocyanins in the final stages of fruit ripening, coinciding with the change in fruit color. Furthermore, the results during the fruit ripening process evidenced that the content of tyrosol and hydroxytyrosol derivatives and tocopherols in the fruit largely determines the content of these groups of compounds in the oil. The information acquired could be useful for the selection of the most suitable moment in the ontogeny of the olive fruit for the search for key genes in the biosynthesis of phenolic compounds.

## 1. Introduction

Virgin olive oil (VOO) is one of the main contributors to the healthy and nutritional properties of the Mediterranean diet due to its content of monounsaturated fatty acids and, especially, its minor components. Among them, the fraction of phenolic compounds has been shown to possess powerful health-promoting antioxidant properties, particularly in relation to cardiovascular diseases, inflammation, cancer, and a general increase in life expectancy [1,2,3]. This fraction comprises both polar phenolic compounds, mostly with secoiridoid structures, and lipophilic phenolic compounds, mainly tocopherols (vitamin E), which have been the subject of different health claims. Thus, evidence of the protective effect of VOO phenolics for cardiovascular diseases has been approved by the European Commission [4], which can be applied to oils containing at least 250 ppm of hydroxytyrosol and derivatives, and on the relationship between dietary intake of vitamin E and protection of DNA, proteins, and lipids from oxidative damage [5].

The presence of secoiridoid derivatives in VOO, containing in their chemical structures the phenolic alcohol tyrosol (*p*-HPEA, Ty) or its hydroxyl derivative hydroxytyrosol (3,4-DHPEA, HTy), is related to the content of phenolic glucosides initially present in the olive fruit and the activity of hydrolytic and oxidative enzymes acting on these glucosides during the oil extraction process [6,7]. The main phenolic glucosides in olive fruit are oleuropein, ligstroside, and demethyloleuropein [8,9], whose enzymatic hydrolysis during the oil extraction process results in the formation of the dialdehydic forms of decarboxymethyloleuropein and decarboxymethylligstroside aglucones (3,4-DHPEA-EDA and *p*-HPEA-EDA, respectively) and the aldehydic forms of oleuropein and ligstroside aglucones (3,4-DHPEA-EA and *p*-HPEA-EA, respectively) [6,7]. Also noteworthy among the phenolics in olive fruit is the fraction of flavonoid derivatives, albeit in much lower concentration, consisting mainly of flavones and anthocyanins.

Olive fruit also contain high amounts of vitamin E. The term vitamin E includes tocopherols and tocotrienols vitamers that are produced exclusively by plants and that are identified as α, β, γ, and δ-forms depending on the number and position of methyl groups in the ring. Only tocopherols are present in olive [10], although Georgiadou et al. [11] found significant content of tocotrienols in VOO. Theoretically, dicotyledonous plants, such as the olive fruit, do not synthesize tocotrienols [12]. Both, secoiridoids and tocopherols, share a common structure composed of a terpenic residue (secoiridoid in the phenolic glucosides and phytol in the tocopherols) and a phenolic residue (tyrosol and hydroxytyrosol in the phenolic glucosides and chromanol in the tocopherols) whose biosynthetic origin is the amino acid tyrosine.

Today, the functional quality of VOO is considered an objective for olive breeding programs given the health benefits of VOO consumption. Since these functional properties reside mainly in their phytochemical content, phenolic compounds are currently used as quality markers of VOO and are already being used as a trait in new breeding programs [13]. However, due to the long juvenile phase and unproductive period, the classical selection process in olive breeding programs can take up to twenty years. The implementation of marker-assisted selection would reduce the time and effort for advancing in the selection process, but the scarce knowledge on the genetic control of the main agronomic traits still limits the progress of olive breeding programs compared to other fruit trees species [14]. In this regards, transcriptomic approaches have been used in recent years to reveal genes associated with important traits, including the biosynthesis of several compounds affecting the functional and organoleptic properties of olive fruit and oil [15]. 

The results obtained so far point out the need to know more precisely how the biosynthesis of these compounds occurs during the ontogeny of the fruit. Therefore, in this study, we aimed to provide further insight into the accumulation dynamics of the main phenolic compounds during the development and ripening of the olive fruit. The information acquired will make it possible to identify more precisely the most advantageous moment in the olive fruit ontogeny for the development of genomic tools that facilitate the search for key genes in the biosynthetic process and the study of their regulation.

## 2. Materials and Methods

### 2.1. Reagents

Reagents for extraction and other measurements were supplied by Sigma-Aldrich Co. (St. Louis, MO, USA). Phenolics were purchased from Extrasynthese (Genay, France) (oleuropein (≥98%), verbascoside (≥98%), tyrosol-1′-*O*-glucoside (≥98%), rutin (≥99%), luteolin-7-*O*-glucoside (≥98%), apigenin-7-*O*–glucoside (≥99%), cyanidin-3-*O*-glucoside (≥97%), cyanidin-3-*O*-rutinoside (≥96%), hydroxytyrosol (≥98%), tyrosol (≥98%), luteolin (≥99%), apigenin (≥99%), pinoresinol (≥95%), acetoxypinoresinol (≥95%)), except for ligstroside (≥98%), which was supplied by ChemFaces (Wuhan, China), and vainillin (≥99%), vanillic acid (≥97%), *p*-coumaric acid (≥98%), cinnamic acid (≥99%), ferulic acid (≥99%), and the standards for quantification (syringic acid (≥95%), *o*-coumaric acid (≥97%) and *p*-hydroxyphenylacetic acid (≥98%)), which were obtained from Sigma Chemical Co. (St. Louis, MO, USA). Non-commercially available demethyloleuropein, hydroxytyrosol-1′-*O*-glucoside, hydroxytyrosol-4-*O*-glucoside, and the main oil secoiridoids derivatives were purified from olive leaves, fruits, and oils by solid-phase extraction (SPE) using C18 cartridges (Supelco, Bellefonte, PA, USA) and high-performance liquid chromatography (HPLC) preparative system. HPLC-grade methanol and acetonitrile were from Panreac (Panreac Applichem, Barcelona, Spain). Solutions were prepared using ultrapure water produced by Milli-Q water purification system (Millipore, MA, USA).

### 2.2. Plant Material

Seven olive cultivars (*Olea europaea* L.) from the Core-36 olive collection of the World Olive Germplasm Bank (WOGB, Cordoba, Spain) established by Belaj et al. [16] (‘Dokkar’, ‘Menya’, ‘Piñonera’, ‘Picual’, ‘Arbequina’, ‘Fishomi’, and ‘Abou kanani’) and three genotypes of the crossing of cultivars Picual × Arbequina (‘UCI-20’, ‘UCI-21’ and ‘UCI-42’) were studied based on their contrasting levels of phenolic compounds and tocopherols in both the fruit and the oil [9,10,17]. Trees, two per genotype, were grown at the experimental orchards of the WOGB (for the assessment of phenotype consistency across the years 2008–2022) and of the Instituto de la Grasa (for the study of the accumulation dynamics of phenolics during the 2020–2021 season), each in the same agronomic and pedoclimatic conditions at 5–6 m spacing and an irrigation regime with drip fertigation from the moment of flowering until the complete ripening of the fruit. Fruit harvest was carried out by hand throughout the fruit development from 3 to 20 weeks after flowering (WAF) and the ripening process. For the latter, harvest dates were related to the maturation index (MI) determined on the basis of the color of the skin and the mesocarp [18]: R-I, olive fruits with green-yellowish epidermis (MI~1); R-II, turning olive fruits, around 50% color (MI~2.5); and R-III, fully colored fruits with white mesocarp (MI~4). During fruit development, samples (20–50 fruits) were collected and used for the extraction of phenolic compounds. The remainder was pitted, frozen in liquid nitrogen, and stored at −80 °C until tocopherol extraction. Sampling during the ripening phase was carried out as above, but an additional 1–2 kg of fruits were used for olive oil extraction. Samples were extracted and analyzed in duplicate.

### 2.3. Olive Oil Extraction

Virgin olive oil was extracted from olive fruits using an Abencor extractor system (Comercial Abengoa, S.A., Seville, Spain), which mimics the industrial process of VOO extraction at laboratory scale. The processing parameters was described by Martínez et al. [19] and consisted of a stainless-steel hammer mill running at 3000 rpm equipped with a 5 mm sieve, a kneader operating at 28 °C for 30 min, and a basket centrifuge working at 3500 rpm for 1 min. The oils were decanted in the dark, paper-filtered, and stored under nitrogen atmosphere at 4 °C until analysis of phenolic compounds and tocopherols. 

### 2.4. Extraction and Analysis of Phenolic Compounds from Olive Fruit and Oil

Fruit phenolic compounds were extracted according to a previously developed protocol [6]. Thin longitudinal pieces of mesocarp tissue were cut from olive fruits with a scalpel and kept at 4 °C for 72 h in dimethyl sulfoxide (6 mL/g FW) containing syringic acid (24 mg/mL) as internal standard. The extracts were filtered through a 0.45 μm Nylon filter and kept at −20 °C until HPLC analysis.

VOO phenolic compounds were isolated by solid-phase extraction (SPE) on a diol-bonded phase cartridge (Supelco, Bellefonte, PA, USA) based on the method by Mateos et al. [20]. A standard solution (0.5 mL) consisting of *p*-hydroxyphenyl-acetic acid (120 µg/mL) and *o*-coumaric acid (10 µg/mL) in methanol was added to each oil sample (2.5 g) in this extraction procedure. 

Both fruit and oil phenolic extracts were analyzed as described by García-Rodríguez et al. [6] in a Beckman Coulter liquid chromatographic system equipped with a diode array detector and a Superspher RP 18 column (4.6 mm i.d. × 250 mm, particle size 4 µm, Dr. Maisch GmbH, Ammerbuch, Germany) at 35 °C. Phenolics were monitored at three different wavelengths 280, 335, and 517 nm and quantified considering the internal standard and calibration curves for each of them. The tentative identification of compounds by their retention times and UV-Vis spectra was confirmed by HPLC/ESI-qTOF-HRMS on a Dionex Ultimate 3000 RS UHPLC liquid chromatograph system (Thermo Fisher Scientific, Waltham, MA, USA) operated for mass analysis using a micrOTOF-QII high-resolution time-of-flight mass spectrometer (UHRTOF) with qQ-TOF geometry (Bruker Daltonics, Bremen, Germany) equipped with an electrospray ionization (ESI) interface and a similar column. 

Fruit phenolics were grouped into the three main groups of compounds: hydroxytyrosol derivatives (HTy-Der), comprising oleuropein, demethyloleuropein, oleuropein aglucone, verbascoside, hydroxytyrosol-1′-*O*-glucoside and hydroxytyrosol-4-*O*-glucoside; tyrosol derivatives (Ty-Der), including ligstroside, ligstroside aglucone, and tyrosol-1′-*O*-glucoside; and flavonoids, containing rutin, luteolin-7-*O*-glucoside, apigenin-7-*O*–glucoside, cyanidin-3-*O*-glucoside, and cyanidin-3-*O*-rutinoside.

Oil phenolics were grouped into five main groups of compounds: hydroxytyrosol derivatives (HTy-Der), including 3,4-DHPEA-DEA, 3,4-DHPEA-EA, HTy, and HTy acetate; tyrosol derivatives (Ty-Der), containing *p*-HPEA-DEA, *p*-HPEA-EA, and Ty; flavonoids, comprising luteolin, and apigenin; lignans, including pinoresinol, and acetoxypinoresinol; and simple phenols, grouping vainillin, vanillic acid, *p*-coumaric acid, cinnamic acid, and ferulic acid.

### 2.5. Extraction and Analysis of Tocopherols from Olive Fruit and Oil

The analysis of tocopherols from olive oil is based on the official IUPAC method [21] with slight modifications. The oil was dissolved in hexane (50 mg oil/mL) that contained α-tocopherol acetate (0.5 mg/mL) as internal standard. After filtration through 0.45 μm Nylon filter, it was analyzed by HPLC in a Beckman-Coulter system equipped with a Jasco FP-1520 fluorescence detector (JASCO Corporation, Tokyo, Japan) and a Tracer LiChrosorb Si 60 column (250 mm × 4.6 mm, 5 μm) (Tecknokroma, Barcelona, Spain). The elution was carried out with an isocratic mixture of hexane: isopropanol (99:1) at a flow rate of 1 mL/min at 35 °C. An excitation wavelength of 290 nm and an emission wavelength of 330 nm were used for detection of compounds. The internal standard α-tocopherol acetate and the response factors calculated for each of the tocopherols (α, β, and γ) were used for quantification. 

Fruit tocopherols were extracted by mixing 50 mg of homogenized tissue in liquid nitrogen with 1 mL of methanol containing α-tocopherol acetate 0.5 mg/mL as internal standard and 0.05% BHT in a 2 mL Eppendorf tube. The mixture was stirred at maximum speed in a Heidolph Multi Reax shaker (Heidolph Instruments GmbH & Co., Schwabach, Germany) for 10 min and filtered through 0.22 µm Nylon filter. The filtrate was stored at −20 °C until HPLC analysis, which was carried out with the HPLC system described above but equipped with a Sunshell C18 column (250 mm × 4 mm, 3.5 µm) (ChromaNik Technologies, Inc., Osaka, Japan). The elution was carried out with an isocratic mixture of methanol/acetonitrile (1:1) at a flow rate of 1 mL/min at 35 °C. Detection and quantitation were performed as described above for oil tocopherols. 

### 2.6. Statistical Analysis

Statistical analysis was performed using Excel 2016 and STATISTICA (Statsoft Inc., Tulsa, OK, USA). Data was subjected to analysis of variance (ANOVA) for multiple mean comparisons of content of fruit and oil phenolics along the ontogeny of the fruit according to the Fisher’s LSD test (*p* < 0.05). Pearson correlations were performed at *p* < 0.001 to study the relationship between the main groups of phenolic compounds from the olive fruit and the oil.

## 3. Results and Discussion

We have previously evidenced that the main phenolics of VOO are secoiridoid derivatives resulting from the hydrolysis during oil extraction process of oleuropein, ligstroside, and demethyloleuropein [9], which are the main phenolic glycosides present in the olive fruit. It has also been found that the content of these phenolics as well as tocopherols in VOO depends largely on the olive cultivar [10]. Based on these data, we have studied the dynamics of the synthesis of phenolic compounds and tocopherols in different olive genotypes from a WOGB core collection selected for having very contrasting content. Furthermore, three genotypes from the progeny of the cross ‘Picual’ × ‘Arbequina’ (‘UCI-20’, ‘UCI-21’ and ‘UCI-42’), previously assessed in terms of phenolic and tocopherol content [10,11,12,13,14,15,16,17] have also been selected for this study due to the contrasting content in phenolic compounds and tocopherols found in the ripe fruits and their oils.

**Figure 1:**
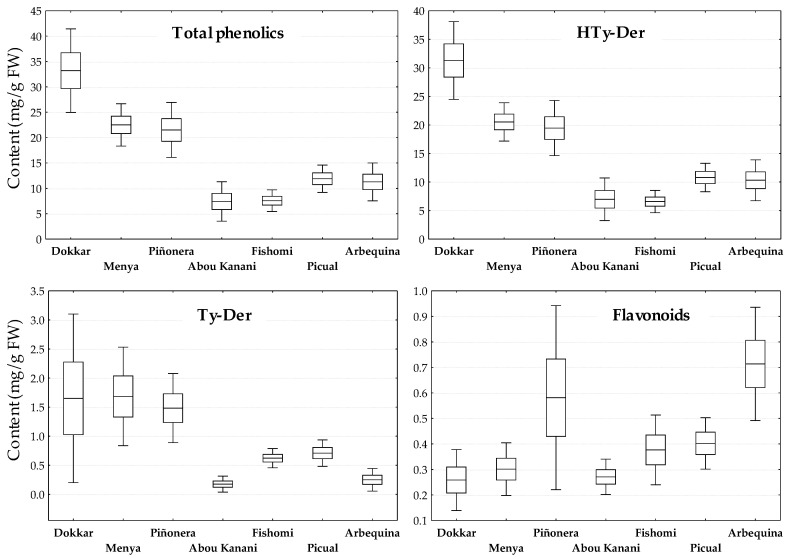
Variability across years for the content (mg/g FW) of the main groups of phenolic compounds in the fruits of selected cultivars from WOGC. Horizontal inner lines in the boxes are mean values. The height in a box is equal to the standard error, and the whiskers are the 0.95 confidence interval. Olive fruits at turning stage (R-II, MI~2,5) were hand-picked over the crop years 2008–2022, and each sample was extracted and analyzed twice for phenolics composition.

In order to evaluate the consistency of their respective phenotypic characters in relation to the phenolic content, these seven cultivars of the WOGB core collection were assessed across different crop seasons (Figure 1). For this purpose, the trees were grown under the same agronomic (i.e., fertilization and irrigation) and edapho-climatic conditions and the fruits were carefully hand-picked at the same ripening stage (turning, R-II) in at least eight different crop years in the period 2008–2022, so that the content of phenolics would only be affected by its crop year. Figure 1 does not contain data on the three genotypes of the cross ‘Picual’ × ‘Arbequina’ (‘UCI-20’, ‘UCI-21’, and ‘UCI-42’) since the study did not cover a long enough period of years. HTy-Der are by far the main contributors to the total content of phenolic compounds in the fruit, followed by the Ty-Der and finally the flavonoids, although in the case of the Arbequina cultivar, the content of flavonoids exceeds that of Ty-Der, constituting a good identification marker of the cultivar. In all cases, it was also found that the phenotype of each cultivar is generally maintained over the different years, clearly marking differences between the selected cultivars, with the exceptions of the Ty-Der content for the high phenolic content cultivars (‘Dokkar’, ‘Menya’ and ‘Piñonera’) and that of flavonoids for cultivars ‘Piñonera’ and ‘Arbequina’, which shows a high variability across the years. Thus, the cultivar ‘Dokkar’ showed to be representative of cultivars high in phenolics (>30 mg/g FW), ‘Menya’ and ‘Piñonera’ of the medium content cultivars (20–25 mg/g FW), and ‘Abou kanani’ and ‘Fishomi’ are representatives of cultivars low in phenolics (<10 mg/g FW). As shown, cultivars ‘Picual’ and ‘Arbequina’ might be considered almost as cultivars low in phenolics.

**Figure 2:**
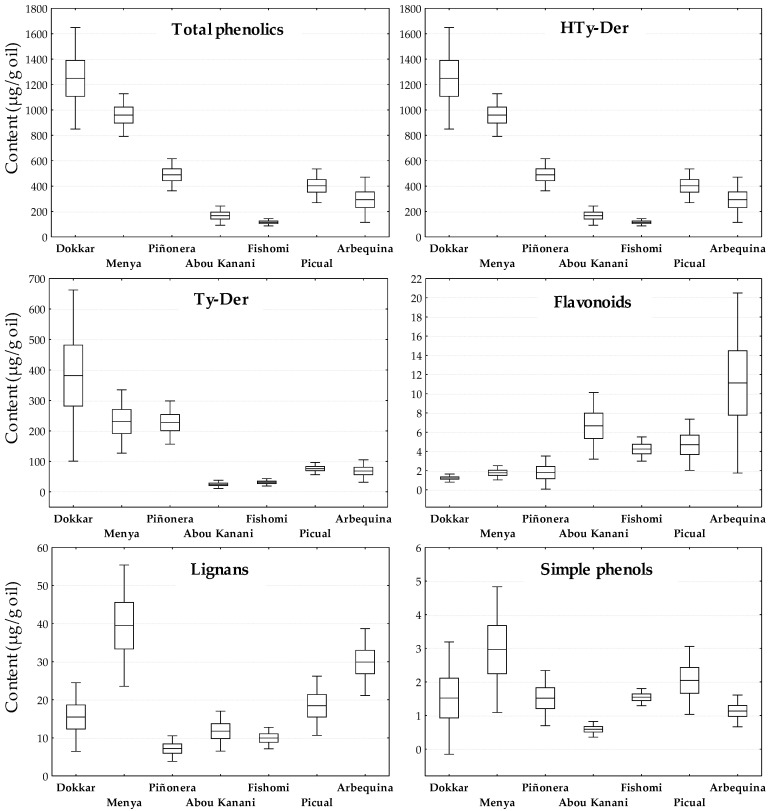
Variability across years for the content (µg/g oil) of the main groups of phenolic compounds in the oils from selected cultivars from WOGC. Horizontal inner lines in the boxes are mean values. The height in a box is equal to the standard error and the whiskers are the 0.95 confidence interval. Olive fruits at turning stage (R-II, MI~2,5) were hand-picked over the crop years 2008–2022 and processed for oil extraction in the same conditions, and each sample was extracted and analyzed twice for phenolics composition.

Similarly, the variability across different crop seasons of the phenolic content of the oils from the selected cultivars has been studied. As shown in Figure 2, the pattern coincides in general with that obtained for the content of fruit phenolic content (Figure 1), although a gap is now observed between the total phenolic content found for the cultivars ‘Menya’ and ‘Piñonera’ towards high and low phenolic content in the oils, respectively. As mentioned above, in addition to the content of phenolic glycosides in the olive fruit, the content of HTy-Der and Ty-Der in the oil is also related to the activity of the hydrolytic and oxidative enzymes acting during the oil extraction process that can substantially modify the final content of these compounds in the oil [6,7,22]. As in the case of the fruit, HTy-Der are the main phenolics in the oil, followed by Ty-Der. Next, the content of flavonoids and lignans are found in approximately the same order of magnitude, while the simple phenols do not reach 0.3% of the phenolic compounds in the oil. Again, the data agree with those found in other studies [9,17]. It was also found in the oil that in all cases the character of each cultivar in terms of phenolic content is generally maintained throughout the years, clearly marking the differences between the selected cultivars. A notable variability across the years is only detected for the content of Ty-Der in the ‘Dokkar’ cultivar and of flavonoids in the ‘Arbequina’ cultivar, which reflects the variability found for these components in the fruits from which they come, albeit to a greater extent. Even so, they remain significantly differentiable from the rest of the cultivars in the content of these compounds.

Regarding tocopherols, we had previously carried out similar studies on both the genetic factor and the variability between years in VOO [10]. It was observed that cultivars such as ‘Dokkar’ or ‘Piñonera’ are representatives of high-tocopherol cultivars, ‘Abou kanani’, ‘Picual’, or ‘Arbequina’ are considered as low-tocopherol cultivars, and ‘Menya’ or ‘Fishomi’ are medium-content cultivars. It should be noted that the cultivar ‘Shengeh’ from that study [10] was later renamed ‘Fishomi’ by the WOGB curator (personal communication). On the other hand, the individuals from the segregating progeny of the cross of ‘Picual’ × ‘Arbequina’, ‘UCI-21’, ‘UCI-42’, and ‘UCI-20’, could be classified into the groups of high, medium, and low tocopherol content in the corresponding oils, respectively.

**Figure 3:**
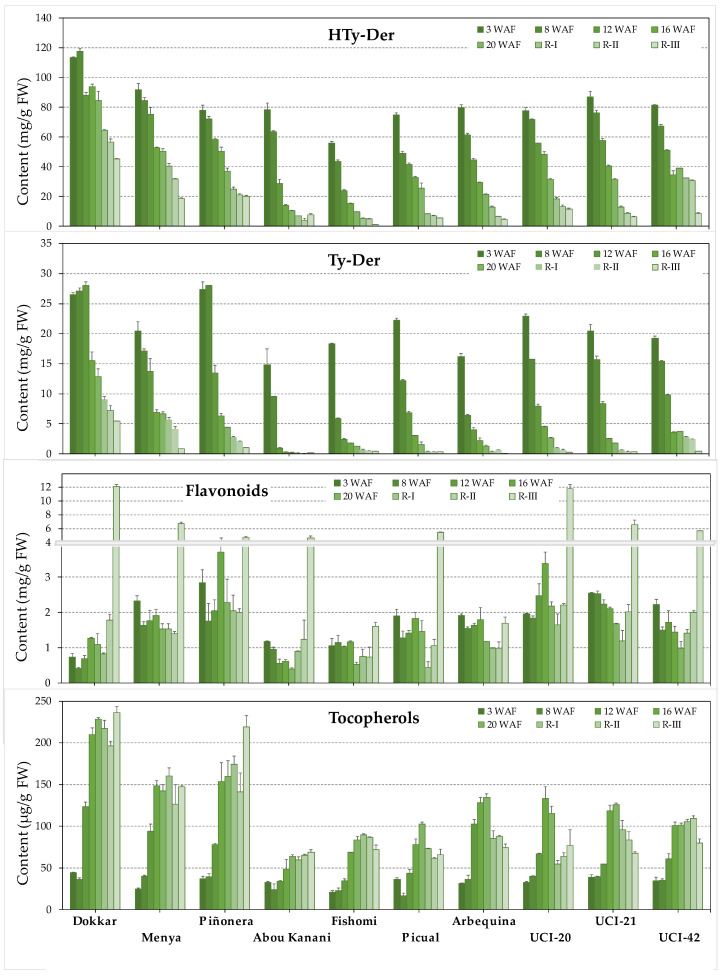
Content of the main groups of phenolic compounds in fruits of seven cultivars and three genotypes from the cross of cultivars ‘Picual’ × ’Arbequina’ along the development and ripening of olive fruit, from 3 weeks after flowering (3 WAF) to fully ripe fruits (R-III). Data are the mean plus standard deviation from two extractions and analyses. The statistical significance of the data is shown in Table S1 of the Supplementary Material.

Once it was proven that the phenotypic character of each genotype is maintained quite well across the years, a study of the biosynthesis dynamics for the main phenolic compounds related to the functionality of the VOO was carried out. It was observed in general that the fruit experiences a dramatic increase in the synthesis of HTy-Der and Ty-Der compounds just after fruit set, at least in the first three weeks after flowering (Figure 3). Malik and Bradford [23] observed that the content of oleuropein, the main compound among the HTy-Der, begins to increase with the expansion of fertilized pistils. The accumulation is so pronounced that in the first two months of fruit life (8 WAF) the content of HTy-Der and Ty-Der compounds represent on average between 5% and 15% of the fresh weight of ‘Fishomi’ and ‘Dokkar’ fruits, respectively. After this strong increase in the content of HTy-Der and Ty-Der, a general tendency to decrease is observed in all the genotypes studied. These results agree with data from the scarce studies that have been carried out in this sense that also show a decrease in the content of some of these compounds along the olive fruit development [24,25,26], and therefore it is assumed that it is a general pattern in olive fruit although with significant differences among the genotypes in relation to the speed at which this decrease occurs.

As shown previously in the snapshot of the content of phenolic compounds in ripe fruits in different years (Figure 1), the phenotype differences between cultivars seem to be maintained throughout the entire process of development and ripening of the fruit, from 3 weeks after flowering (3 WAF) to fully ripe fruits (R-III). Thus, ‘Dokkar’ fruits have the highest content of HTy-Der and Ty-Der compounds on average, while the ‘Fishomi’ and ‘Abou kanani’ fruits show the lowest content among those studied. Apparently, the later the onset of the typical decrease in the content of these compounds occurs throughout the development of the fruit, the greater the probability that the fruit will reach the ripening phase with higher phenolic content and, consequently, with a greater possibility to give rise to oils with a higher phenolic content.

Among the HTy-Der and Ty-Der compounds, the content of oleuropein and ligstroside shows a pattern of evolution similar to that of these total phenolics in the fruit, although the typical decrease in content after the peak of synthesis occurs more markedly in the case of the ligstroside (Supplementary Material, Table S1). As for demethyloleuropein, it was only found significantly in the ripening phase in the cultivars with the highest content of phenolic compounds (‘Dokkar’, ‘Menya’, and ‘Piñonera’) and in the ‘Arbequina’ cultivar, becoming the major phenolic compound in their fruits in the last stage of ripening (R-III). This phenolic compound, which probably comes from oleuropein as maintained by Servili et al. [27], could also serve as a cultivar marker since it is only found in some cultivars [28,29]. Curiously, the three genotypes of the ‘Picual’ × ‘Arbequina’ progeny have inherited the character of the Picual parent, presenting very low levels of demethyloleuropein along the ripening phase.

Also, among the HTy-Der compounds, verbascoside stands out, which, as in the case of demethyloleuropein, can constitute an olive cultivar marker. Verbascoside was found in large quantities in cultivars ‘Dokkar’ (3.65 mg/g FW as average of all stages of fruit development and ripening) and ‘Piñonera’ (1.84 mg/g FW as average of all stages), as well as in the ‘Arbequina’ cultivar to a lesser extent (1.02 mg/g FW as average of all stages). Furthermore, it was observed that ‘UCI-21’ has inherited the phenotype of the ‘Arbequina’ parent, being the genotype displaying the highest content of all under study (5.44 mg/g FW as average of all stages). The verbascoside content increases steadily throughout the process of fruit development in all the genotypes until reaching a maximum within 12–16 WAF. From here, the content of this compound decreases during the ripening phase, except for a slight increase at the last ripening stage. This pattern coincides with what was observed in different olive cultivars including ‘Picual’ and ‘Arbequina’ [23,30].

The pattern of flavonoid content during the fruit development and ripening is different from that found for HTy-Der and Ty-Der compounds (Figure 3: Flavonoids), more closely resembling that of the accumulation of verbascoside described above. Two well-differentiated phases are distinguished in the synthesis of these compounds. On one hand, the synthesis of flavones (rutin, luteolin-7-*O*-glucoside, and apigenin-7-*O*-glucoside) throughout the ontogeny of the fruit, but especially during fruit development, and, on the other, the exponential synthesis of anthocyanins (cyanidin-3-*O*-glucoside and cyanidin-3-*O*-rutinoside) occurring at the end of the ripening phase were reflected in the change in coloration of the fruit from green towards dark-purple. Although flavones also have a specific peak, albeit minor, of active synthesis just after fruit set, subsequently, a maximum of flavone synthesis is generally observed around 16 WAF. Then, a decrease in the total flavone content occurs during the ripening phase. Among the genotypes studied in this work, ‘Piñonera’ stands out for being a high producer of flavones. It should be noted that the three genotypes from the ‘Picual’ × ‘Arbequina’ cross presented higher flavone content than their parents. On the other hand, unlike what happens in relation to the content of HTy-Der and Ty-Der compounds, ‘Dokkar’ appears as a cultivar with a low level of flavones. However, this cultivar actively synthesizes anthocyanins during the ripening phase, being, together with ‘UCI-20’, the genotypes that accumulate the most anthocyanins among those studied (1.58 and 1.40 mg/g FW as average of the ripening phase, respectively). In all cases, the content of cyanidin-3-*O*-rutinoside was much higher than that of cyanidin-3-*O*-glucoside (20 times higher on average), in full agreement with what was found for other cultivars [30,31,32]. Their content is only detectable when the fruit enters the ripening phase, with the highest levels found in stage R-III, as might be expected from the development of the fruit color.

Pérez et al. [10] found that tocopherol content in oils also preserves the character over the years in different genotypes. The range of tocopherol content in the fruit, expressed as an average in all the stages studied during fruit development and ripening, was between the cultivar ‘Abou kanani’, which has the lowest tocopherol content (49.64 µg/g PF), and the ‘Dokkar’ cultivar that presents the highest level (161.63 µg/g PF). The content of tocopherols displays a very different profile from that of the main phenolic compounds during the developmental and ripening processes of the olive fruit (Figure 3: Tocopherols). In general, it is characterized by a constant increase in the synthesis throughout fruit development to reach a maximum accumulation at the onset of fruit ripening, around 20 WAF. However, Georgiadou et al. [11,33] did not find major changes in the tocopherol content of the fruit of ‘Koroneiki’ cultivar during this period. The pattern of tocopherol content and the variability during the ripening phase of the genotypes under study (average of ripening stages, 64.67–216.81 µg/g FW) was very similar to that observed by Georgiadou et al. [34] in five Greek olive cultivars. In general, after the peak at the onset of fruit ripening, a decrease in the tocopherol content occur during this phase, except for a specific increase observed in half of the genotypes at the final stage of the ripening phase, when the fruit has just completed its coloration (stage R-III). This phenomenon was already observed by Georgiadou et al. [11,34] in different Greek olive cultivars at the same ripening stage (ca. 28–30 WAF), which they suggested to be due to a reflective physiological transition in olive fruits. 

**Figure 4:**
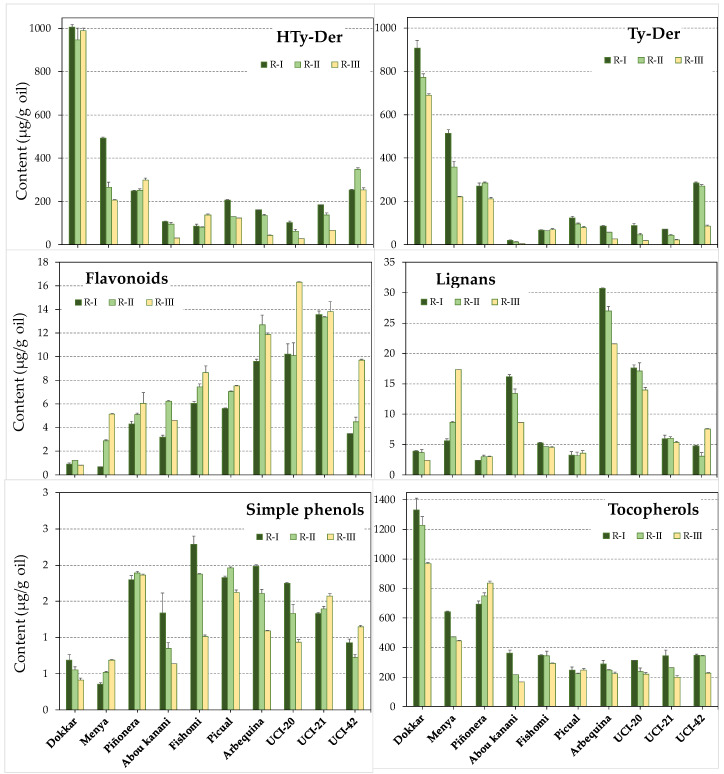
Content (µg/g oil) of the main groups of phenolic compounds of oils from seven cultivars and three genotypes from the cross of cultivars ‘Picual’ × ’Arbequina’ along the fruit ripening phase. Data are the mean plus standard deviation from two extractions and analyses. The statistical significance of the data is shown in Table S2 of the Supplementary Material.

In order to study the dynamics of the content of phenolic compounds and tocopherols in the oils and the relationship with the content of these compounds in the fruits, oils were extracted and analyzed at the three selected ripening stages: beginning of ripening (R-I), turning fruits (R-II), and fruits with fully colored skin (R-III). In general, the content of phenolic compounds in the oil decreases along the ripening phase (Supplementary Materials, Table S2). This trend is common to the majority of the olive cultivars studied to date [7,35]. As shown in Figure 4, this is essentially owing to the pattern followed by both the HTy-Der and Ty-Der content, since these compounds together represent an average of 95% (70.6% and 24.1%, respectively) of the oil phenolic fraction in all stages of fruit ripening. 

On the contrary, the dynamics of the flavonoid content, which represents 2.8% (expressed as average of all stages) of the phenolics in the oil, shows a tendency to increase during fruit ripening. On the other hand, the content of lignans (2.2% as the average of all stages) and the sum of simple phenols (0.3% as the average of all stages) tend to decrease in most of the genotypes (Figure 4). Regarding tocopherols in the oil, the data suggest that the evolution of the content throughout the ripening of the fruit is a fairly conserved process. In general terms, a decrease is observed in most cultivars, except for cultivar ‘Piñonera’ that displays a characteristic increase. This general tendency towards a decrease in the oil tocopherol content during fruit ripening was already observed for most of the olive cultivars [10,36,37]. 

**Figure 5:**
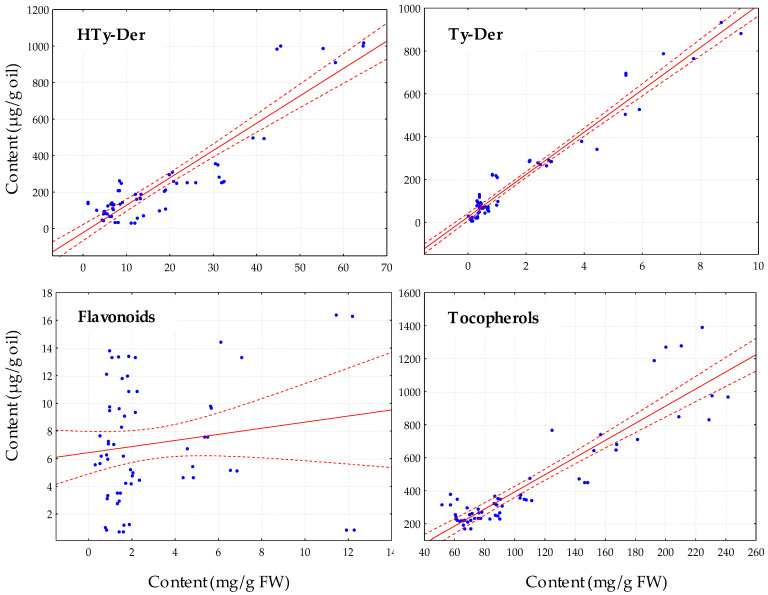
Comparison of the content of the main groups of phenolic compounds in the fruit and the oil samples from seven cultivars and three genotypes from the cross of cultivars ‘Picual’ × ’Arbequina’ along the fruit ripening phase. The regression line (solid red) and the 95% confidence interval for the regression line (dashed red lines) are represented.

It is observed at first sight that, in general, genotypes rich in phenolic content in the fruit will give rise to oils with high phenolic content. Thus, a Pearson correlation coefficient (*r*) of 0.946 (*p* < 0.001) has been calculated for the relationship between the content of phenolic compounds in the fruit and the oil during the ripening phase. This coefficient is higher than that calculated in a previous study with olive cultivars from the WOGB Core-36 collection [9], which included the cultivars of the present study. As would be expected, the best correlation was obtained between the content of HTy-Der (*r* = 0.907, *p* < 0.001) and Ty-Der (*r* = 0.975, *p* < 0.001) in the fruit and in the oil (Figure 5). However, a poor non-significant correlation has been found between the flavonoid content in the fruit and in the oil (*r* = 0.157, *p* > 0.1), which suggests that the flavonoids comprising the phenolic fraction of the oils might not come from the hydrolysis of their corresponding glycosylated derivatives in the fruit, but more probably from the free flavone fraction of the fruit. The high levels of anthocyanins in the fruit, especially in the last stage of the ripening phase (R-III), also do not have any reflection in the final composition of the main phenolic compounds of the oil. It is likely that there is no glycosidase activity capable of breaking the sugar bond of flavonoids during the oil extraction process. However, there is experimental evidence of the participation of a specific β-glucosidase activity, whose coding genes have also been identified, that transforms the HTy-Der and Ty-Der compounds of the fruit during the oil extraction process [7].

As for the tocopherols, a strong relationship has also been found between the content of these metabolites in the fruit and in the oil (Figure 5: Tocopherols), displaying a Pearson correlation coefficient of 0.909 (*p* < 0.001). To our knowledge, this is the first time the relationship between the tocopherol content of the fruit and the oil has been studied.

## 4. Conclusions

It has been proven that the phenotypic differences, relative to the content of the most important phenolic compounds from a quantitative and functional point of view, of these genotypes are maintained over the years in the turning fruit (R-II) and that these differences are also conserved in the different stages of development and ripening throughout the ontogeny of the olive fruit, which ultimately translate into significant differences in the phenolic composition of the oils produced by these genotypes. Even with these differences, all genotypes share just after fruit set an intense increase in the synthesis of HTy-Der and Ty-Der compounds, by far the main phenolic compounds of the olive fruit, as well as a subsequent constant decrease in the content of these compounds from about two months after fruit set until the end of the ripening phase. The accumulation dynamics of flavonoids and tocopherols are different from that of HTy-Der and Ty-Der compounds, presenting a synthesis peak just before the onset of fruit ripening. Furthermore, within the flavonoids, all the genotypes also share a strong increase in the synthesis and accumulation of anthocyanins that occurs in the final stages of fruit ripening and results in a change in fruit color. In addition, this work sheds light on the relationship that exists between the phenolic fraction of the olive fruit along the ripening process and its corresponding oil. All these results could be useful for marker-assisted breeding programs, facilitating the selection of the most appropriate moment in the ontogeny of the olive fruit for the development of genomic tools and study of key genes in the biosynthesis of its main phenolic compounds.

## Data Availability

Data are contained within the article and Supplementary Material.

## Supplementary Materials

The following are available online at https://www.mdpi.com/article/10.3390/antiox13010012/s1: Table S1: Content of the main phenolic compounds (µg/g FW), individual and groups of compounds, in olive fruits of seven cultivars and three genotypes from the cross of cultivars ‘Picual’ × ’Arbequina’ along the development and ripening. Fruit harvest was carried out by hand throughout the fruit development from 3 to 20 weeks after flowering (WAF) and the ripening process: R-I, olive fruits with green-yellowish epidermis (MI = 1); R-II, turning olive fruits, around 50% color (MI = 2.5); and R-III, fully colored fruits with white mesocarp (MI = 4). Data are the mean plus/minus standard deviation from two extractions and analyses. Table S2: Content of the main the phenolic compounds (µg/g oil), individual and groups of compounds, in olive oils extracted from seven cultivars and three genotypes from the cross of cultivars ‘Picual’ × ’Arbequina’ along the ripening. Fruit harvest was carried out by hand throughout the fruit ripening process: R-I, olive fruits with green-yellowish epidermis (MI = 1); R-II, turning olive fruits, around 50% color (MI = 2.5); and R-III, fully colored fruits with white mesocarp (MI = 4). Data are the mean plus/minus standard deviation from two extractions and analyses.
