# Supplementary material for: Accumulation Patterns of Metabolites Responsible for the Functional Quality of Virgin Olive Oil during Olive Fruit Ontogeny
Source: Antioxidants (Basel). 2023 Dec 20;13(1):12. doi: 10.3390/antiox13010012 (PMC10812685; doi:10.3390/antiox13010012)
Supplement: Supplementary file 1 [file antioxidants-13-00012-s001.zip › antioxidants-2762598-supplementary.pdf]

Table S1. Content of the main the phenolic compounds (μg/g FW), individual and groups of compounds, in olive fruits of seven cultivars and three genotypes from the cross of cultivars 'Picual' x 'Arbequina' along the development and ripening. Fruit harvest was carried out by hand throughout the fruit development from 3 to 20 weeks after flowering (WAF) and the ripening process: R-I, olive fruits with green-yellowish epidermis (MI = 1); R-II, turning olive fruits, around 50% color (MI = 2.5); and R-III, fully colored fruits with white mesocarp (MI = 4). Data are the mean plus-minus standard deviation from two extractions and analyses.

Genotype / stage	hydroxytyrosol-1′-O -glucoside + hydroxytyrosol-4-O -glucoside	hydroxytyrosol-4-O -glucoside	demethyloleuropein	oleuropein	ligstroside	oleuropein aglycone	ligstroside aglycone	verbascoside	rutin	luteolin-7-O -glucoside	apigenin-7-O -glucoside	cyanidin-3-O -glucoside	cyanidin-3-O -rutinoside	α-tocopherol	β-tocopherol	γ-tocopherol	Hydroxytyrosol derivatives (HTy-Der)	Tyrosol derivatives (Ty-Der)	Flavonoids	Tocopherols
**Dokkar 3 WAF**	1527.54 ± 49.40	743.21 ± 7.45	18.76 ± 1.46	111245.68 ± 115.79	25607.55 ± 294.34	307.62 ± 72.18	151.25 ± 0.98	614.37 ± 6.05	639.69 ± 85.25	84.50 ± 19.32	12.54 ± 3.49	0.00 ± 0.00	0.00 ± 0.00	41.58 ± 0.60	0.45 ± 0.05	2.33 ± 0.21	113713.98 ± 241.97	26502.01 ± 300.81	736.74 ± 108.06	44.36 ± 0.45
**Dokkar 8 WAF**	1561.20 ± 43.01	1424.72 ± 7.89	10.32 ± 0.17	113427.28 ± 2137.95	25563.40 ± 521.15	288.71 ± 11.48	113.35 ± 14.63	2544.14 ± 8.35	328.13 ± 21.21	82.68 ± 2.29	6.64 ± 0.05	0.00 ± 0.00	0.00 ± 0.00	34.73 ± 1.44	0.36 ± 0.13	1.29 ± 0.11	117831.66 ± 2114.60	27101.47 ± 498.63	417.45 ± 23.45	36.37 ± 1.68
**Dokkar 12 WAF**	2180.83 ± 142.43	671.54 ± 2.40	10.59 ± 0.59	79643.12 ± 1541.40	26950.15 ± 740.07	721.88 ± 410.16	376.16 ± 150.75	5554.58 ± 47.01	509.67 ± 47.92	168.38 ± 40.10	6.69 ± 3.00	0.00 ± 0.00	0.00 ± 0.00	120.63 ± 4.66	0.48 ± 0.02	2.55 ± 1.00	88111.01 ± 1855.56	27997.85 ± 586.92	684.73 ± 91.01	123.65 ± 5.64
**Dokkar 16 WAF**	2505.37 ± 323.47	166.51 ± 10.65	35.21 ± 1.29	82548.58 ± 2595.23	14582.79 ± 1367.09	4068.00 ± 537.51	750.25 ± 50.74	4634.87 ± 43.65	866.53 ± 16.27	323.80 ± 40.92	75.68 ± 7.09	0.00 ± 0.00	0.00 ± 0.00	206.28 ± 8.18	0.37 ± 0.03	3.32 ± 0.07	93792.03 ± 1779.18	15499.55 ± 1428.48	1266.01 ± 31.74	209.97 ± 8.22
**Dokkar 20 WAF**	1627.93 ± 78.93	149.50 ± 5.93	30.14 ± 17.92	70515.67 ± 5410.05	11500.26 ± 1204.13	7209.94 ± 613.86	1234.50 ± 83.30	5055.95 ± 437.11	772.29 ± 244.78	241.13 ± 50.15	76.12 ± 21.55	0.00 ± 0.00	0.00 ± 0.00	222.59 ± 1.80	0.39 ± 0.11	5.34 ± 0.26	84439.63 ± 6400.02	12884.26 ± 1281.50	1089.53 ± 316.48	228.32 ± 2.17
**Dokkar R-I**	1285.48 ± 4.66	91.26 ± 4.67	9.63 ± 3.75	52199.22 ± 349.77	7858.75 ± 414.05	7554.50 ± 234.26	1118.97 ± 69.42	3628.26 ± 39.81	529.77 ± 40.49	232.11 ± 9.72	54.85 ± 1.25	0.00 ± 0.00	0.00 ± 0.00	211.06 ± 9.45	0.71 ± 0.30	5.77 ± 0.04	64677.09 ± 146.91	9068.98 ± 488.14	816.73 ± 29.53	217.55 ± 9.71
**Dokkar R-II**	1286.71 ± 55.33	116.85 ± 10.75	2137.32 ± 66.12	41521.52 ± 2228.25	6011.46 ± 718.51	8206.04 ± 483.30	1129.53 ± 7.78	3621.49 ± 142.10	392.62 ± 4.58	152.54 ± 39.38	32.09 ± 6.08	321.94 ± 42.80	878.29 ± 76.03	189.64 ± 5.21	0.78 ± 0.11	6.12 ± 0.11	56773.08 ± 2008.50	7257.84 ± 737.04	1777.48 ± 168.86	196.54 ± 5.43
**Dokkar R-III**	1610.15 ± 33.87	191.22 ± 4.58	33943.82 ± 557.57	5872.05 ± 1040.00	5236.75 ± 21.65	205.55 ± 1.51	21.19 ± 20.12	3532.00 ± 62.99	492.85 ± 87.49	151.51 ± 27.98	28.81 ± 7.38	2533.58 ± 67.46	8899.97 ± 312.25	230.36 ± 7.64	1.06 ± 0.04	4.91 ± 0.14	45163.58 ± 580.81	5449.15 ± 6.12	12106.71 ± 256.87	236.33 ± 7.74
**Menya 3 WAF**	581.90 ± 0.93	448.05 ± 28.36	22.61 ± 4.14	90333.52 ± 4635.25	19668.40 ± 1603.70	691.15 ± 164.75	299.44 ± 47.90	69.11 ± 9.13	1803.34 ± 221.49	382.75 ± 86.73	141.38 ± 17.59	0.00 ± 0.00	0.00 ± 0.00	22.34 ± 1.27	0.35 ± 0.01	1.76 ± 0.04	91698.29 ± 4456.30	20415.89 ± 1584.16	2327.48 ± 152.36	24.44 ± 1.29
**Menya 8 WAF**	1421.39 ± 30.90	1148.59 ± 39.86	42.26 ± 1.48	82523.38 ± 2087.18	15698.35 ± 350.34	534.82 ± 89.13	245.70 ± 25.76	20.32 ± 0.83	1073.41 ± 77.58	486.51 ± 36.11	70.47 ± 1.68	0.00 ± 0.00	0.00 ± 0.00	38.95 ± 1.16	0.37 ± 0.06	0.68 ± 0.08	84542.18 ± 2209.52	17092.64 ± 415.96	1630.39 ± 115.37	39.99 ± 1.02
**Menya 12 WAF**	417.47 ± 185.74	197.30 ± 60.35	43.17 ± 1.66	74160.63 ± 4547.11	13319.43 ± 2110.92	444.72 ± 131.56	234.62 ± 56.48	63.61 ± 9.45	1146.74 ± 182.63	543.86 ± 103.32	80.21 ± 0.71	0.00 ± 0.00	0.00 ± 0.00	92.81 ± 8.44	0.51 ± 0.00	0.98 ± 0.09	75129.60 ± 4485.14	13751.35 ± 2107.05	1770.81 ± 286.66	94.30 ± 8.36
**Menya 16 WAF**	758.68 ± 27.10	347.84 ± 10.09	61.80 ± 4.35	51218.46 ± 398.98	6250.32 ± 495.74	761.34 ± 65.51	279.27 ± 11.30	242.64 ± 4.87	1242.17 ± 130.66	538.12 ± 23.36	134.22 ± 17.01	0.00 ± 0.00	0.00 ± 0.00	144.09 ± 5.71	1.46 ± 0.23	3.21 ± 0.23	53042.92 ± 297.15	6877.42 ± 474.35	1914.51 ± 171.03	148.76 ± 5.72
**Menya 20 WAF**	726.50 ± 30.03	161.59 ± 13.86	53.18 ± 3.67	49268.24 ± 1757.56	6402.01 ± 350.93	125.87 ± 37.33	109.71 ± 15.85	322.67 ± 23.06	1023.30 ± 111.69	408.25 ± 28.23	97.27 ± 11.94	0.00 ± 0.00	0.00 ± 0.00	137.93 ± 6.64	1.48 ± 0.08	2.98 ± 0.51	50496.46 ± 1769.65	6673.31 ± 348.93	1528.82 ± 151.86	142.39 ± 7.23
**Menya R-I**	726.50 ± 30.03	161.59 ± 13.86	53.18 ± 3.67	39268.24 ± 1757.56	5402.01 ± 350.93	125.87 ± 37.33	109.71 ± 15.85	322.67 ± 23.06	1023.30 ± 111.69	408.25 ± 28.23	97.27 ± 11.94	0.00 ± 0.00	0.00 ± 0.00	154.98 ± 9.39	2.10 ± 0.11	2.97 ± 0.73	40496.46 ± 1769.65	5673.31 ± 348.93	1528.82 ± 151.86	160.05 ± 10.23
**Menya R-II**	752.42 ± 64.46	82.55 ± 14.07	597.48 ± 28.33	28939.35 ± 249.31	3977.22 ± 344.03	788.11 ± 110.76	124.42 ± 6.34	629.53 ± 55.93	686.04 ± 45.95	331.13 ± 16.13	56.29 ± 2.88	14.24 ± 0.72	310.64 ± 9.38	122.39 ± 22.64	1.40 ± 0.01	2.62 ± 0.54	31706.90 ± 452.13	4184.20 ± 364.44	1398.33 ± 54.86	126.41 ± 23.16
**Menya R-III**	1129.96 ± 24.15	105.13 ± 6.86	12327.46 ± 42.75	4826.15 ± 287.94	705.47 ± 26.33	85.64 ± 25.60	38.65 ± 14.58	526.23 ± 6.73	431.91 ± 8.87	212.32 ± 8.01	19.32 ± 2.60	235.22 ± 15.79	5811.23 ± 221.14	142.85 ± 0.78	1.58 ± 0.03	3.12 ± 0.53	18895.44 ± 202.17	849.26 ± 4.90	6709.99 ± 217.44	147.56 ± 1.28
**Piñonera 3 WAF**	1176.18 ± 39.56	1052.22 ± 22.36	28.26 ± 1.43	76649.90 ± 3363.96	26188.80 ± 1266.42	119.13 ± 10.30	127.56 ± 7.00	65.35 ± 1.51	2474.57 ± 329.67	334.22 ± 32.06	34.54 ± 5.05	0.00 ± 0.00	0.00 ± 0.00	34.10 ± 2.18	0.47 ± 0.07	2.55 ± 0.42	78038.82 ± 3393.14	27368.59 ± 1281.78	2843.32 ± 366.78	37.12 ± 2.67
**Piñonera 8 WAF**	533.94 ± 55.91	1057.86 ± 43.31	27.35 ± 31.22	70866.31 ± 1568.45	26686.23 ± 67.89	387.41 ± 32.01	256.45 ± 15.52	517.72 ± 42.23	1383.56 ± 456.22	343.43 ± 25.63	29.68 ± 13.84	0.00 ± 0.00	0.00 ± 0.00	38.66 ± 3.64	0.29 ± 0.01	0.53 ± 0.04	72332.73 ± 1582.91	28000.54 ± 9.06	1756.67 ± 495.69	39.48 ± 3.67
**Piñonera 12 WAF**	2242.25 ± 127.49	984.08 ± 206.79	65.27 ± 13.22	53288.60 ± 113.62	12269.17 ± 962.92	323.37 ± 267.23	180.72 ± 129.24	2415.05 ± 116.32	1529.21 ± 289.28	481.93 ± 18.06	35.50 ± 11.15	0.00 ± 0.00	0.00 ± 0.00	76.84 ± 1.00	0.35 ± 0.12	0.60 ± 0.24	58334.55 ± 611.45	13433.98 ± 1298.95	2046.64 ± 318.50	77.79 ± 1.36
**Piñonera 16 WAF**	1947.49 ± 150.44	574.00 ± 43.17	145.83 ± 29.84	45833.62 ± 2236.04	5679.87 ± 368.26	74.77 ± 13.93	42.69 ± 39.94	2487.17 ± 232.10	3310.04 ± 811.33	283.22 ± 64.53	124.90 ± 35.06	0.00 ± 0.00	0.00 ± 0.00	151.82 ± 23.14	0.47 ± 0.09	0.95 ± 0.04	50488.88 ± 2662.35	6296.56 ± 451.37	3718.17 ± 910.93	153.24 ± 23.09
**Piñonera 20 WAF**	918.72 ± 74.35	228.93 ± 14.73	101.01 ± 18.42	33692.17 ± 1928.35	4122.88 ± 25.71	30.03 ± 20.67	83.91 ± 25.47	2391.18 ± 80.08	2072.77 ± 609.72	146.91 ± 33.01	63.83 ± 28.25	0.00 ± 0.00	0.00 ± 0.00	158.22 ± 18.84	0.52 ± 0.21	1.19 ± 0.23	37133.11 ± 2080.53	4435.72 ± 14.48	2283.52 ± 670.97	159.93 ± 18.82
**Piñonera R-I**	1186.68 ± 81.18	234.41 ± 16.52	914.29 ± 10.02	20329.36 ± 1006.70	2553.82 ± 89.18	48.25 ± 6.03	5.17 ± 0.95	2567.80 ± 268.45	1280.07 ± 276.26	342.49 ± 120.18	43.55 ± 16.50	49.99 ± 6.00	334.91 ± 21.54	171.13 ± 9.85	1.43 ± 0.05	2.07 ± 0.11	25046.38 ± 1360.32	2793.40 ± 104.75	2051.01 ± 440.48	174.63 ± 9.69
**Piñonera R-II**	844.64 ± 28.83	162.70 ± 3.69	1901.59 ± 94.22	16399.55 ± 274.31	1945.01 ± 8.17	231.18 ± 1.89	24.83 ± 3.72	1869.36 ± 104.00	1163.67 ± 41.66	312.19 ± 3.70	45.04 ± 2.97	86.55 ± 10.66	400.61 ± 42.71	139.00 ± 22.78	0.42 ± 0.02	1.68 ± 0.13	21246.31 ± 503.24	2132.54 ± 8.20	2008.07 ± 94.31	141.10 ± 22.93
**Piñonera R-III**	1935.39 ± 18.96	375.17 ± 7.27	11450.67 ± 80.63	4451.54 ± 445.53	596.38 ± 53.38	22.64 ± 14.77	27.50 ± 15.19	2425.19 ± 6.16	1203.93 ± 44.91	309.16 ± 46.21	41.38 ± 0.44	181.80 ± 14.64	2963.75 ± 75.91	214.97 ± 13.76	1.68 ± 0.05	2.36 ± 0.29	20285.42 ± 524.19	999.06 ± 30.92	4700.02 ± 182.12	219.01 ± 14.00
**Abou kanani 3 WAF**	370.66 ± 476.42	450.91 ± 116.92	213.75 ± 42.07	77460.49 ± 5234.33	14215.05 ± 2340.61	422.83 ± 382.28	164.36 ± 222.61	61.81 ± 11.23	821.49 ± 10.68	311.12 ± 14.50	47.22 ± 6.53	0.00 ± 0.00	0.00 ± 0.00	28.31 ± 0.40	0.42 ± 0.08	4.05 ± 0.63	78529.55 ± 4406.46	14830.33 ± 2680.14	1179.83 ± 10.35	32.78 ± 0.95
**Abou kanani 8 WAF**	615.04 ± 9.07	516.62 ± 8.02	13.79 ± 5.53	62435.99 ± 361.06	8796.56 ± 37.08	634.96 ± 10.98	221.63 ± 8.04	47.42 ± 4.37	479.26 ± 48.99	442.25 ± 13.64	28.37 ± 1.07	0.00 ± 0.00	0.00 ± 0.00	23.88 ± 6.70	0.00 ± 0.00	0.00 ± 0.00	63747.20 ± 350.91	9534.81 ± 37.10	949.88 ± 63.69	23.88 ± 6.70
**Abou kanani 12 WAF**	259.22 ± 53.51	57.26 ± 10.05	79.87 ± 6.58	26833.69 ± 2432.71	788.86 ± 110.38	1501.97 ± 353.55	71.43 ± 15.99	33.26 ± 3.68	232.29 ± 0.94	320.18 ± 103.80	12.17 ± 4.23	0.00 ± 0.00	0.00 ± 0.00	33.10 ± 1.41	0.24 ± 0.05	0.45 ± 0.06	28708.02 ± 2829.51	917.56 ± 136.42	564.64 ± 108.96	33.79 ± 1.29
**Abou kanani 16 WAF**	168.29 ± 9.36	16.21 ± 1.56	75.32 ± 6.22	13621.88 ± 669.64	256.15 ± 32.29	8.16 ± 0.13	13.59 ± 7.19	36.47 ± 1.48	223.70 ± 29.74	368.71 ± 19.17	21.52 ± 0.52	0.00 ± 0.00	0.00 ± 0.00	48.31 ± 12.10	0.09 ± 0.13	0.30 ± 0.42	13910.13 ± 683.87	285.95 ± 41.04	613.92 ± 49.44	48.70 ± 11.54
**Abou kanani 20 WAF**	277.42 ± 14.58	3.34 ± 2.30	63.25 ± 5.56	10228.42 ± 305.66	193.84 ± 87.75	14.22 ± 0.07	3.73 ± 0.43	26.13 ± 1.22	134.02 ± 34.72	250.96 ± 9.10	12.08 ± 2.07	0.00 ± 0.00	0.00 ± 0.00	63.41 ± 1.65	0.23 ± 0.01	0.30 ± 0.03	10609.44 ± 326.95	200.92 ± 89.62	397.05 ± 27.68	63.94 ± 1.61
**Abou kanani R-I**	258.41 ± 6.65	6.19 ± 0.14	24.35 ± 29.70	6630.91 ± 127.93	145.06 ± 7.48	57.08 ± 3.27	2.14 ± 0.06	54.31 ± 0.23	100.62 ± 21.01	177.20 ± 5.78	9.00 ± 0.37	14.56 ± 0.31	597.50 ± 0.57	58.73 ± 3.13	0.31 ± 0.03	0.78 ± 0.07	7025.06 ± 101.39	153.40 ± 7.27	898.87 ± 14.74	59.82 ± 3.23
**Abou kanani R-II**	314.62 ± 151.03	7.18 ± 2.05	22.02 ± 25.94	3548.50 ± 1024.86	84.45 ± 35.14	52.06 ± 65.82	21.08 ± 11.41	94.40 ± 41.65	101.89 ± 32.34	155.09 ± 42.56	7.92 ± 2.39	10.20 ± 3.36	958.25 ± 464.71	64.12 ± 1.11	0.33 ± 0.02	0.61 ± 0.06	4031.60 ± 1177.66	112.71 ± 25.78	1233.35 ± 545.36	65.06 ± 1.03
**Abou kanani R-III**	1315.28 ± 94.03	8.21 ± 0.13	5.83 ± 4.10	6170.29 ± 468.38	123.12 ± 8.62	2.89 ± 1.93	31.81 ± 20.54	324.76 ± 13.77	192.95 ± 28.64	315.60 ± 17.99	13.50 ± 0.05	37.95 ± 0.46	4061.60 ± 343.64	68.14 ± 2.90	0.30 ± 0.00	0.69 ± 0.10	7819.05 ± 570.15	163.14 ± 29.29	4621.60 ± 333.41	69.13 ± 2.80
**Fishomi 3 WAF**	130.03 ± 1.89	205.75 ± 1.41	42.17 ± 50.77	55586.17 ± 1124.59	17891.21 ± 108.48	234.12 ± 75.33	209.22 ± 8.98	10.51 ± 0.32	890.59 ± 161.92	137.44 ± 28.42	32.14 ± 13.66	0.00 ± 0.00	0.00 ± 0.00	16.48 ± 1.45	0.42 ± 0.09	3.96 ± 0.50	56003.00 ± 1146.94	18306.18 ± 116.05	1060.17 ± 203.99	20.87 ± 2.03
**Fishomi 8 WAF**	213.05 ± 5.24	258.75 ± 8.47	34.97 ± 4.72	42732.74 ± 1051.47	5514.04 ± 83.34	590.40 ± 23.06	121.53 ± 5.14	0.00 ± 0.00	880.59 ± 186.70	180.95 ± 2.33	87.36 ± 19.67	0.00 ± 0.00	0.00 ± 0.00	23.03 ± 2.56	0.00 ± 0.00	0.00 ± 0.00	43571.16 ± 1074.00	5894.32 ± 80.01	1148.90 ± 204.04	23.03 ± 2.56
**Fishomi 12 WAF**	186.45 ± 19.35	121.15 ± 26.00	36.74 ± 0.16	23083.33 ± 389.01	2180.17 ± 125.44	768.67 ± 202.10	111.61 ± 42.91	3.68 ± 0.52	614.81 ± 22.76	302.09 ± 1.26	113.20 ± 12.05	0.00 ± 0.00	0.00 ± 0.00	33.57 ± 2.52	0.34 ± 0.02	0.40 ± 0.11	24078.87 ± 610.10	2412.92 ± 194.35	1030.09 ± 9.45	34.31 ± 2.65
**Fishomi 16 WAF**	580.74 ± 24.07	115.86 ± 1.75	65.23 ± 3.82	14276.47 ± 451.11	1658.63 ± 28.28	60.18 ± 5.45	42.97 ± 1.30	170.12 ± 11.66	703.99 ± 27.98	315.29 ± 53.25	143.74 ± 10.23	0.00 ± 0.00	0.00 ± 0.00	67.47 ± 0.05	0.44 ± 0.02	0.91 ± 0.00	15152.75 ± 417.02	1817.46 ± 27.83	1163.03 ± 35.49	68.81 ± 0.07
**Fishomi 20 WAF**	744.63 ± 11.14	61.55 ± 4.12	57.68 ± 1.32	8687.84 ± 184.01	1196.85 ± 59.33	9.73 ± 9.88	4.33 ± 1.36	253.87 ± 4.15	320.95 ± 86.74	160.93 ± 23.42	40.36 ± 3.10	0.00 ± 0.00	0.00 ± 0.00	82.46 ± 4.53	0.32 ± 0.02	0.75 ± 0.19	9753.75 ± 179.81	1262.73 ± 56.57	522.23 ± 66.41	83.54 ± 4.31
**Fishomi R-I**	949.43 ± 178.78	61.75 ± 14.27	25.00 ± 23.42	4221.40 ± 303.53	559.90 ± 102.32	50.84 ± 59.36	7.68 ± 0.80	104.44 ± 12.91	305.60 ± 111.32	206.28 ± 36.83	46.38 ± 14.33	12.36 ± 4.11	178.44 ± 47.41	88.10 ± 0.82	0.79 ± 0.20	1.03 ± 0.07	5351.10 ± 412.44	629.33 ± 117.39	749.06 ± 214.00	89.92 ± 1.09
**Fishomi R-II**	909.25 ± 21.24	63.29 ± 1.94	5.08 ± 2.44	4009.27 ± 340.16	426.73 ± 11.79	6.36 ± 1.01	23.26 ± 29.08	37.88 ± 7.97	313.75 ± 128.08	209.58 ± 82.19	56.97 ± 36.27	15.72 ± 3.28	144.98 ± 18.65	85.07 ± 0.58	0.69 ± 0.04	1.07 ± 0.16	4967.83 ± 352.00	513.27 ± 19.22	741.01 ± 268.47	86.83 ± 0.39
**Fishomi R-III**	903.68 ± 15.75	39.17 ± 1.72	32.45 ± 30.10	149.52 ± 19.46	367.36 ± 13.67	22.34 ± 2.74	12.83 ± 1.14	142.42 ± 3.55	261.59 ± 63.17	117.07 ± 25.58	29.11 ± 13.95	76.24 ± 0.06	1122.94 ± 10.94	71.20 ± 4.76	0.37 ± 0.11	0.58 ± 0.21	1250.41 ± 32.69	419.36 ± 16.52	1606.95 ± 113.70	72.16 ± 5.08
**Picual 3 WAF**	514.37 ± 40.22	548.45 ± 48.58	37.10 ± 32.02	73703.69 ± 1294.63	21355.75 ± 261.53	566.00 ± 218.80	328.87 ± 73.27	16.75 ± 3.62	1550.90 ± 143.15	278.07 ± 47.01	69.77 ± 3.57	0.00 ± 0.00	0.00 ± 0.00	27.37 ± 1.00	0.41 ± 0.04	8.43 ± 0.82	74837.91 ± 1525.25	22233.06 ± 383.38	1898.74 ± 193.73	36.21 ± 1.86
**Picual 8 WAF**	663.02 ± 15.02	910.73 ± 3.13	146.26 ± 28.90	47369.29 ± 1652.75	10998.04 ± 163.82	753.43 ± 78.53	278.17 ± 22.53	0.00 ± 0.00	695.62 ± 100.42	534.06 ± 68.39	57.75 ± 11.90	0.00 ± 0.00	0.00 ± 0.00	15.68 ± 3.29	0.24 ± 0.05	0.83 ± 0.10	48932.00 ± 1618.14	12186.94 ± 144.42	1287.43 ± 180.71	16.76 ± 3.24
**Picual 12 WAF**	1425.60 ± 51.80	812.50 ± 88.29	191.14 ± 31.85	39174.48 ± 908.42	5793.79 ± 166.45	765.08 ± 82.13	231.96 ± 27.22	61.52 ± 6.78	616.47 ± 21.35	730.53 ± 38.65	72.40 ± 6.34	0.00 ± 0.00	0.00 ± 0.00	42.95 ± 4.29	0.33 ± 0.01	0.47 ± 0.10	41617.82 ± 1067.43	6838.25 ± 281.96	1419.40 ± 66.34	43.76 ± 4.40
**Picual 16 WAF**	690.32 ± 2.62	234.01 ± 4.29	286.54 ± 35.19	30530.21 ± 753.22	2801.44 ± 38.54	597.07 ± 7.69	61.70 ± 28.66	649.63 ± 34.81	775.86 ± 36.12	949.11 ± 124.73	102.05 ± 18.69	0.00 ± 0.00	0.00 ± 0.00	77.37 ± 6.59	0.31 ± 0.01	0.49 ± 0.04	32753.78 ± 758.68	3097.15 ± 5.59	1827.02 ± 179.54	78.17 ± 6.64
**Picual 20 WAF**	760.74 ± 154.01	62.54 ± 23.14	205.24 ± 52.44	23857.54 ± 2798.96	1502.39 ± 381.12	12.84 ± 10.24	17.83 ± 17.85	941.29 ± 196.55	666.39 ± 96.88	732.29 ± 190.90	65.90 ± 18.64	0.00 ± 0.00	0.00 ± 0.00	101.16 ± 1.93	0.61 ± 0.01	0.95 ± 0.14	25777.64 ± 3191.72	1582.76 ± 386.41	1464.58 ± 306.42	102.72 ± 2.06
**Picual R-I**	397.10 ± 35.04	9.25 ± 3.17	5.76 ± 4.83	7561.91 ± 173.84	376.26 ± 2.78	28.00 ± 0.13	26.33 ± 3.87	332.00 ± 15.80	195.45 ± 55.79	233.01 ± 101.53	7.72 ± 6.05	0.00 ± 0.00	0.00 ± 0.00	67.51 ± 1.10	2.11 ± 0.08	3.22 ± 0.01	8324.78 ± 229.38	411.84 ± 4.26	436.18 ± 163.37	72.84 ± 1.19
**Picual R-II**	494.37 ± 36.41	32.41 ± 4.56	48.78 ± 66.43	6183.44 ± 166.42	290.15 ± 15.44	17.92 ± 0.71	5.61 ± 1.46	341.35 ± 34.79	317.93 ± 66.72	269.34 ± 100.88	7.42 ± 6.14	107.70 ± 6.02	356.00 ± 5.05	57.17 ± 0.77	1.79 ± 0.06	2.72 ± 0.01	7085.86 ± 162.36	328.17 ± 18.54	1058.39 ± 184.82	61.68 ± 0.73
**Picual R-III**	652.06 ± 11.57	48.62 ± 1.66	45.96 ± 12.97	4474.63 ± 47.06	290.91 ± 49.06	12.91 ± 3.73	6.56 ± 1.36	566.00 ± 21.64	197.35 ± 43.11	195.90 ± 53.41	5.31 ± 3.51	1602.82 ± 65.56	3445.96 ± 135.45	61.65 ± 5.99	1.68 ± 0.45	2.57 ± 0.05	5751.56 ± 30.55	346.09 ± 46.04	5447.34 ± 100.98	65.90 ± 6.38
**Arbequina 3 WAF**	473.47 ± 86.44	369.74 ± 27.59	160.81 ± 42.84	78540.06 ± 2381.59	15592.32 ± 504.14	473.63 ± 249.21	235.99 ± 50.76	77.11 ± 11.23	1285.75 ± 12.49	512.12 ± 16.38	123.23 ± 7.30	0.00 ± 0.00	0.00 ± 0.00	27.21 ± 0.93	0.30 ± 0.03	3.61 ± 0.01	79725.08 ± 2250.44	16198.05 ± 527.31	1921.10 ± 36.17	31.11 ± 0.88
**Arbequina 8 WAF**	1199.94 ± 1.17	701.75 ± 44.81	34.61 ± 32.73	59705.60 ± 1167.06	5670.32 ± 105.49	251.68 ± 5.22	71.81 ± 9.94	280.74 ± 10.42	791.06 ± 53.88	695.54 ± 15.50	62.07 ± 2.82	0.00 ± 0.00	0.00 ± 0.00	34.70 ± 4.94	0.34 ± 0.14	0.99 ± 0.08	61472.57 ± 1206.18	6443.89 ± 160.25	1548.66 ± 41.20	36.03 ± 5.16
**Arbequina 12 WAF**	544.59 ± 59.93	253.32 ± 39.96	86.25 ± 0.55	42319.08 ± 966.68	3761.89 ± 203.19	272.69 ± 72.90	41.66 ± 20.26	1502.15 ± 81.14	800.13 ± 13.42	766.97 ± 76.98	64.74 ± 6.11	0.00 ± 0.00	0.00 ± 0.00	101.93 ± 5.07	0.24 ± 0.04	0.56 ± 0.01	44724.77 ± 1018.93	4056.86 ± 263.41	1631.83 ± 69.67	102.73 ± 5.12
**Arbequina 16 WAF**	507.77 ± 10.64	75.17 ± 6.97	139.07 ± 22.20	26789.37 ± 273.87	2146.08 ± 408.27	15.93 ± 18.28	3.89 ± 1.52	2117.67 ± 35.42	730.26 ± 193.26	943.20 ± 111.89	122.59 ± 42.58	0.00 ± 0.00	0.00 ± 0.00	127.46 ± 6.18	0.29 ± 0.04	0.43 ± 0.05	29569.82 ± 302.57	2225.15 ± 416.76	1796.05 ± 347.74	128.18 ± 6.16
**Arbequina 20 WAF**	790.07 ± 12.24	21.60 ± 0.22	1745.17 ± 66.91	17090.95 ± 89.37	1259.57 ± 179.98	15.64 ± 9.93	10.23 ± 5.58	2028.67 ± 207.98	500.48 ± 8.01	638.44 ± 5.87	33.15 ± 3.43	0.00 ± 0.00	0.00 ± 0.00	134.03 ± 4.06	0.37 ± 0.01	0.40 ± 0.03	21670.49 ± 232.76	1291.40 ± 174.61	1172.07 ± 10.45	134.81 ± 4.02
**Arbequina R-I**	601.76 ± 40.42	3.44 ± 1.16	7638.06 ± 490.96	3560.12 ± 7.31	419.47 ± 17.51	33.45 ± 0.55	4.23 ± 2.44	1031.46 ± 43.43	414.19 ± 16.33	535.07 ± 5.79	34.32 ± 0.05	0.00 ± 0.00	6.45 ± 0.80	84.53 ± 9.40	0.28 ± 0.03	0.34 ± 0.03	12864.85 ± 566.95	427.14 ± 16.23	990.02 ± 9.69	85.14 ± 9.46
**Arbequina R-II**	501.79 ± 7.61	6.84 ± 0.35	5503.28 ± 210.30	8.04 ± 1.28	679.45 ± 16.92	26.78 ± 0.23	10.83 ± 0.17	569.44 ± 22.65	316.54 ± 33.03	494.70 ± 131.14	30.74 ± 9.43	13.45 ± 0.64	129.14 ± 0.21	87.30 ± 1.22	0.31 ± 0.03	0.52 ± 0.00	6609.33 ± 223.83	697.12 ± 17.10	984.57 ± 174.46	88.13 ± 1.25
**Arbequina R-III**	493.78 ± 21.75	13.01 ± 2.44	3579.39 ± 209.82	23.97 ± 14.31	10.45 ± 0.75	20.51 ± 0.12	7.59 ± 4.32	561.35 ± 6.74	226.03 ± 21.83	342.96 ± 68.33	13.18 ± 2.36	27.78 ± 3.82	1089.05 ± 70.36	73.44 ± 4.59	0.37 ± 0.04	0.52 ± 0.07	4679.00 ± 167.14	31.06 ± 6.02	1699.00 ± 166.70	74.33 ± 4.56
**UCI-20 3 WAF**	490.19 ± 61.47	575.57 ± 20.42	19.07 ± 6.80	75922.41 ± 2341.30	21696.77 ± 468.29	1277.92 ± 502.08	659.04 ± 171.30	103.21 ± 54.76	1211.00 ± 33.64	584.02 ± 19.74	171.80 ± 0.98	0.00 ± 0.00	0.00 ± 0.00	29.32 ± 0.80	0.41 ± 0.12	2.99 ± 0.25	77812.80 ± 1839.13	22931.39 ± 317.41	1966.83 ± 14.88	32.72 ± 1.17
**UCI-20 8 WAF**	1822.15 ± 15.62	1688.35 ± 2.60	91.46 ± 6.05	65871.85 ± 20.20	14000.15 ± 48.55	130.96 ± 8.79	75.82 ± 6.87	4064.80 ± 188.51	989.27 ± 15.13	759.35 ± 36.67	92.93 ± 4.95	0.00 ± 0.00	0.00 ± 0.00	39.04 ± 0.53	0.24 ± 0.03	0.55 ± 0.07	71981.22 ± 167.53	15764.32 ± 44.28	1841.55 ± 56.75	39.83 ± 0.57
**UCI-20 12 WAF**	1401.66 ± 149.74	412.18 ± 20.61	123.09 ± 9.09	47563.80 ± 17.06	7457.66 ± 286.74	181.78 ± 15.28	58.87 ± 11.10	6601.88 ± 112.51	1333.74 ± 120.32	1021.59 ± 184.15	125.43 ± 25.22	0.00 ± 0.00	0.00 ± 0.00	66.24 ± 0.85	0.38 ± 0.17	0.58 ± 0.11	55872.20 ± 220.82	7928.72 ± 318.45	2480.75 ± 329.69	67.20 ± 0.56
**UCI-20 16 WAF**	1309.17 ± 8.12	150.51 ± 9.49	480.59 ± 6.77	38185.72 ± 1111.41	4311.69 ± 61.65	10.06 ± 3.24	29.43 ± 36.88	8503.18 ± 621.93	1412.67 ± 46.97	1771.75 ± 334.77	200.65 ± 37.49	0.00 ± 0.00	0.00 ± 0.00	132.34 ± 14.28	0.39 ± 0.02	0.62 ± 0.11	48488.72 ± 1735.23	4491.63 ± 108.02	3385.07 ± 325.29	133.34 ± 14.37
**UCI-20 20 WAF**	478.18 ± 1.71	29.40 ± 2.32	352.42 ± 33.08	23345.99 ± 378.53	2607.59 ± 56.89	5.31 ± 2.18	16.02 ± 10.46	7387.75 ± 419.40	953.78 ± 40.44	1124.97 ± 68.87	105.68 ± 3.98	0.00 ± 0.00	0.00 ± 0.00	114.63 ± 8.38	0.36 ± 0.13	0.59 ± 0.18	31569.64 ± 764.38	2653.01 ± 69.67	2184.44 ± 113.29	115.58 ± 8.69
**UCI-20 R-I**	526.55 ± 35.02	9.95 ± 0.24	12.91 ± 2.30	11899.41 ± 711.47	1001.41 ± 36.08	34.26 ± 24.56	10.98 ± 1.12	5907.23 ± 226.04	641.46 ± 140.25	934.77 ± 156.72	62.17 ± 14.64	0.00 ± 0.00	10.10 ± 1.48	54.20 ± 4.19	0.23 ± 0.01	0.35 ± 0.03	18380.36 ± 950.27	1022.34 ± 37.44	1648.50 ± 310.13	54.78 ± 4.22
**UCI-20 R-II**	971.52 ± 113.53	33.50 ± 0.95	17.29 ± 21.10	7525.36 ± 620.05	605.52 ± 92.07	26.39 ± 27.05	12.52 ± 2.37	4794.86 ± 320.84	628.46 ± 73.17	797.34 ± 29.94	55.02 ± 0.08	22.13 ± 2.50	706.22 ± 12.27	62.77 ± 4.32	0.46 ± 0.06	0.61 ± 0.03	13335.43 ± 1006.27	651.53 ± 90.65	2209.18 ± 52.92	63.84 ± 4.34
**UCI-20 R-III**	2705.50 ± 212.59	112.28 ± 18.19	2.24 ± 0.46	2701.68 ± 45.55	154.73 ± 29.01	16.73 ± 1.42	25.35 ± 2.80	6126.76 ± 393.08	607.60 ± 12.91	701.47 ± 99.24	44.96 ± 3.16	59.69 ± 3.08	10418.59 ± 413.15	75.28 ± 18.05	0.35 ± 0.02	1.29 ± 0.75	11552.91 ± 559.16	292.36 ± 13.61	11832.31 ± 531.53	76.92 ± 18.82
**UCI-21 3 WAF**	1206.04 ± 202.82	820.06 ± 83.45	192.45 ± 29.95	83846.74 ± 4142.50	18961.52 ± 1140.68	1618.49 ± 363.12	672.14 ± 111.29	33.60 ± 0.67	1700.36 ± 38.50	647.30 ± 26.81	198.80 ± 12.86	0.00 ± 0.00	0.00 ± 0.00	29.27 ± 2.96	0.61 ± 0.17	8.90 ± 0.53	86897.32 ± 4012.81	20453.72 ± 1112.84	2546.46 ± 24.55	38.78 ± 3.32
**UCI-21 8 WAF**	903.66 ± 11.98	982.03 ± 8.30	110.76 ± 3.19	74626.23 ± 1656.74	14532.31 ± 500.86	620.75 ± 114.36	200.63 ± 36.96	14.16 ± 1.31	1200.08 ± 55.93	1184.74 ± 29.47	144.70 ± 1.05	0.00 ± 0.00	0.00 ± 0.00	36.81 ± 1.19	0.24 ± 0.01	2.30 ± 0.25	76275.55 ± 1761.00	15714.97 ± 546.12	2529.52 ± 84.34	39.35 ± 0.93
**UCI-21 12 WAF**	1680.99 ± 130.98	677.94 ± 68.40	137.16 ± 0.67	51320.95 ± 1592.41	7011.40 ± 286.57	3615.83 ± 96.44	666.86 ± 17.06	902.93 ± 64.90	1023.28 ± 13.13	1102.88 ± 85.38	114.42 ± 20.13	0.00 ± 0.00	0.00 ± 0.00	54.23 ± 0.07	0.26 ± 0.04	0.37 ± 0.08	57657.86 ± 1562.72	8356.19 ± 337.90	2240.58 ± 118.64	54.86 ± 0.05
**UCI-21 16 WAF**	818.36 ± 18.40	81.92 ± 1.00	136.91 ± 1.80	37632.03 ± 564.84	2373.85 ± 7.60	1767.37 ± 129.46	86.57 ± 28.75	264.94 ± 24.10	934.04 ± 143.15	1059.70 ± 89.78	114.41 ± 4.12	0.00 ± 0.00	0.00 ± 0.00	117.25 ± 6.61	0.28 ± 0.10	0.88 ± 0.39	40619.61 ± 479.68	2542.33 ± 22.16	2108.16 ± 49.24	118.41 ± 7.10
**UCI-21 20 WAF**	922.83 ± 5.17	35.64 ± 3.85	63.75 ± 70.84	29873.98 ± 713.17	1774.47 ± 8.80	446.28 ± 26.25	24.66 ± 6.84	353.77 ± 11.95	699.27 ± 50.90	890.78 ± 22.67	85.50 ± 5.37	0.00 ± 0.00	0.00 ± 0.00	124.40 ± 1.79	0.31 ± 0.08	1.21 ± 0.11	31660.61 ± 685.69	1834.77 ± 11.79	1675.55 ± 22.86	125.92 ± 1.98
**UCI-21 R-I**	781.75 ± 0.34	12.85 ± 2.90	8.95 ± 1.60	11579.25 ± 955.89	635.19 ± 47.46	220.64 ± 19.67	13.33 ± 8.73	186.50 ± 0.96	428.42 ± 70.80	717.02 ± 208.25	42.35 ± 19.62	0.00 ± 0.00	4.98 ± 1.42	94.44 ± 11.09	0.56 ± 0.08	1.15 ± 0.14	12777.09 ± 975.85	661.37 ± 53.29	1192.78 ± 300.10	96.15 ± 11.03
**UCI-21 R-II**	866.37 ± 31.64	27.31 ± 4.06	6.07 ± 3.70	7241.93 ± 421.83	344.61 ± 37.79	565.83 ± 5.23	6.21 ± 1.45	160.60 ± 12.54	317.66 ± 15.75	514.01 ± 120.15	30.65 ± 6.46	82.94 ± 9.19	1066.67 ± 65.03	81.12 ± 9.36	0.71 ± 0.18	1.53 ± 0.33	8840.81 ± 386.58	378.13 ± 32.28	2011.93 ± 216.59	83.35 ± 9.87
**UCI-21 R-III**	1493.42 ± 3.31	47.80 ± 0.65	31.72 ± 30.82	4669.79 ± 315.07	277.25 ± 13.32	50.67 ± 3.32	22.40 ± 14.08	222.13 ± 14.39	300.99 ± 52.83	323.21 ± 59.06	14.86 ± 7.12	319.70 ± 41.61	5644.10 ± 756.17	65.28 ± 1.46	0.90 ± 0.20	1.29 ± 0.21	6467.72 ± 305.27	347.45 ± 1.41	6602.85 ± 678.77	67.47 ± 1.87
**UCI-42 3 WAF**	509.91 ± 41.50	566.46 ± 21.50	28.24 ± 7.41	80331.14 ± 652.69	18398.33 ± 432.92	521.52 ± 188.04	298.53 ± 52.80	24.21 ± 0.36	1442.44 ± 89.92	621.92 ± 45.69	167.86 ± 8.94	0.00 ± 0.00	0.00 ± 0.00	31.06 ± 3.51	0.34 ± 0.06	3.09 ± 0.67	81415.03 ± 430.20	19263.33 ± 358.63	2232.22 ± 144.55	34.49 ± 4.24
**UCI-42 8 WAF**	736.38 ± 40.21	924.42 ± 27.77	50.24 ± 1.06	66164.79 ± 729.28	14294.61 ± 120.70	532.62 ± 91.30	205.47 ± 29.58	6.21 ± 2.85	843.11 ± 54.25	593.95 ± 27.68	68.81 ± 4.20	0.00 ± 0.00	0.00 ± 0.00	34.14 ± 1.16	0.33 ± 0.14	0.81 ± 0.22	67490.23 ± 776.46	15424.50 ± 122.50	1505.87 ± 86.13	35.27 ± 1.51
**UCI-42 12 WAF**	2025.70 ± 169.85	1263.71 ± 56.56	86.07 ± 16.17	46126.07 ± 348.28	7922.04 ± 47.54	1957.33 ± 143.85	612.58 ± 4.20	890.71 ± 19.91	880.30 ± 181.73	783.45 ± 129.28	59.74 ± 12.84	0.00 ± 0.00	0.00 ± 0.00	60.44 ± 5.76	0.24 ± 0.03	0.43 ± 0.27	51085.86 ± 318.54	9798.32 ± 99.91	1723.49 ± 323.84	61.11 ± 6.00
**UCI-42 16 WAF**	769.18 ± 33.45	238.87 ± 6.55	38.07 ± 10.14	31825.00 ± 2541.63	3163.67 ± 77.44	995.43 ± 196.76	154.97 ± 72.76	1052.50 ± 13.22	700.70 ± 147.47	688.54 ± 1.05	61.27 ± 10.24	0.00 ± 0.00	0.00 ± 0.00	99.86 ± 4.73	0.31 ± 0.07	0.57 ± 0.22	34680.18 ± 2748.48	3557.51 ± 143.66	1450.50 ± 158.77	100.73 ± 4.44
**UCI-42 20 WAF**	735.18 ± 67.20	82.19 ± 15.76	84.81 ± 16.16	36745.14 ± 140.16	3644.44 ± 9.28	9.11 ± 4.75	9.01 ± 2.19	1360.12 ± 91.01	505.00 ± 161.32	437.92 ± 20.61	32.87 ± 6.04	0.00 ± 0.00	9.77 ± 0.30	100.14 ± 2.01	0.31 ± 0.08	0.79 ± 0.29	38934.36 ± 29.47	3735.64 ± 8.67	985.56 ± 187.67	101.24 ± 2.39
**UCI-42 R-I**	457.71 ± 16.19	37.96 ± 2.56	11.61 ± 0.75	30903.32 ± 142.61	2785.49 ± 58.32	36.42 ± 47.13	10.77 ± 1.11	1076.05 ± 11.46	606.73 ± 3.55	581.93 ± 93.33	31.18 ± 3.55	9.51 ± 1.26	180.73 ± 6.16	104.87 ± 2.81	0.31 ± 0.10	0.53 ± 0.02	32485.12 ± 67.07	2834.22 ± 56.87	1410.08 ± 107.86	105.71 ± 2.69
**UCI-42 R-II**	428.06 ± 7.83	23.58 ± 0.77	11.68 ± 0.11	29334.39 ± 351.75	2433.96 ± 58.59	22.52 ± 14.87	10.09 ± 0.57	1081.52 ± 19.13	767.69 ± 0.77	845.82 ± 14.02	55.97 ± 0.59	15.93 ± 3.48	323.64 ± 25.95	108.04 ± 3.34	0.33 ± 0.03	0.71 ± 0.23	30878.15 ± 393.69	2467.63 ± 59.93	2009.04 ± 42.09	109.08 ± 3.60
**UCI-42 R-III**	822.63 ± 129.73	62.86 ± 3.94	6.83 ± 1.03	6798.16 ± 113.23	380.23 ± 16.82	400.91 ± 294.62	15.00 ± 17.30	634.23 ± 2.16	269.83 ± 30.81	216.79 ± 21.37	9.70 ± 1.92	210.71 ± 10.48	4966.64 ± 23.16	78.61 ± 5.09	0.50 ± 0.10	0.70 ± 0.03	8662.77 ± 312.25	458.08 ± 4.42	5673.67 ± 20.47	79.80 ± 5.23

Table S2. Content of the main the phenolic compounds (μg/g oil), individual and groups of compounds, in olive oils extracted from seven cultivars and three genotypes from the cross of cultivars 'Picual' x 'Arbequina' along the ripening. Fruit harvest was carried out by hand throughout the fruit ripening process: R-I, olive fruits with green-yellowish epidermis (MI = 1); R-II, turning olive fruits, around 50% color (MI = 2.5); and R-III, fully colored fruits with white mesocarp (MI = 4). Data are the mean plus-minus standard deviation from two extractions and analyses.

Genotype / stage	hidroxytyrosol (HTy)	tyrosol (Ty)	vanillic acid	vainillin	*p*-coumaric acid	hidroxytyrosol acetate	3,4-DHPEA-DEA (oleacein)	*p*-HPEA-DEA (oleocanthal)	pinoresinol	cinnamic acid	acetoxypinoresinol	3,4-DHPEA-EA (oleuropein aglycone)	*p*-HPEA-EA (ligstroside aglycon)	ferulic acid	luteolin	apigenin	α-tocopherol	β-tocopherol	γ-tocopherol	Hydroxytyrosol derivatives (HTy-Der)	Tyrosol derivatives (Ty-Der)	Flavonoids	Lignans	Simple phenols	Tocopherols
**Dokkar R-I**	11.16 ± 0.41	7.29 ± 0.55	0.11 ± 0.03	0.20 ± 0.02	0.19 ± 0.04	1.04 ± 0.19	48.09 ± 0.10	36.43 ± 20.14	1.13 ± 0.08	0.18 ± 0.11	2.74 ± 0.00	945.90 ± 11.82	863.07 ± 16.21	0.02 ± 0.00	0.67 ± 0.09	0.23 ± 0.02	1311.62 ± 77.49	5.53 ± 0.55	16.08 ± 1.01	1006.18 ± 12.14	906.79 ± 36.90	0.90 ± 0.11	3.87 ± 0.08	0.69 ± 0.08	1333.22 ± 79.05
**Dokkar R-II**	13.13 ± 0.58	7.36 ± 0.28	0.13 ± 0.01	0.13 ± 0.01	0.20 ± 0.00	1.02 ± 0.04	104.08 ± 3.50	112.04 ± 1.49	1.40 ± 0.63	0.07 ± 0.02	2.21 ± 0.07	829.00 ± 51.11	653.86 ± 14.56	0.02 ± 0.00	0.92 ± 0.03	0.27 ± 0.01	1202.40 ± 56.49	5.05 ± 0.52	20.55 ± 2.48	947.23 ± 55.23	773.26 ± 16.33	1.19 ± 0.04	3.61 ± 0.56	0.55 ± 0.04	1228.01 ± 59.49
**Dokkar R-III**	4.07 ± 0.07	2.92 ± 0.17	0.10 ± 0.03	0.12 ± 0.00	0.13 ± 0.00	10.28 ± 0.13	667.71 ± 37.37	519.93 ± 2.39	1.70 ± 0.13	0.04 ± 0.00	0.62 ± 0.16	309.07 ± 26.35	167.43 ± 4.87	0.02 ± 0.00	0.63 ± 0.02	0.18 ± 0.02	954.81 ± 4.23	7.23 ± 0.52	7.67 ± 0.39	991.13 ± 11.08	690.27 ± 7.43	0.82 ± 0.00	2.32 ± 0.02	0.41 ± 0.03	969.70 ± 4.35
**Menya R-I**	6.25 ± 1.64	15.13 ± 1.24	0.07 ± 0.05	0.17 ± 0.01	0.01 ± 0.00	1.17 ± 0.36	7.42 ± 0.52	47.88 ± 0.89	0.99 ± 0.08	0.09 ± 0.02	4.58 ± 0.29	479.29 ± 1.18	451.05 ± 17.26	0.01 ± 0.00	0.53 ± 0.01	0.15 ± 0.00	625.31 ± 0.15	0.75 ± 1.07	16.15 ± 1.39	494.13 ± 3.71	514.06 ± 16.91	0.68 ± 0.01	5.58 ± 0.37	0.35 ± 0.02	642.22 ± 2.60
**Menya R-II**	12.44 ± 2.22	13.78 ± 2.04	0.25 ± 0.03	0.15 ± 0.01	0.03 ± 0.01	1.53 ± 0.13	38.54 ± 3.80	65.18 ± 9.27	0.98 ± 0.08	0.06 ± 0.00	7.68 ± 0.23	212.35 ± 21.84	279.53 ± 18.80	0.03 ± 0.00	2.52 ± 0.10	0.32 ± 0.03	461.48 ± 1.99	0.78 ± 0.07	8.98 ± 0.26	264.85 ± 23.29	358.49 ± 26.03	2.84 ± 0.13	8.67 ± 0.15	0.51 ± 0.01	471.24 ± 1.66
**Menya R-III**	7.18 ± 0.22	11.59 ± 0.54	0.31 ± 0.00	0.13 ± 0.01	0.16 ± 0.00	12.32 ± 0.17	90.88 ± 0.38	110.04 ± 1.69	1.74 ± 0.05	0.07 ± 0.01	15.57 ± 0.01	95.28 ± 3.57	100.12 ± 0.13	0.03 ± 0.00	4.76 ± 0.01	0.37 ± 0.03	435.48 ± 2.82	0.32 ± 0.45	11.09 ± 2.82	205.66 ± 3.55	221.76 ± 2.37	5.12 ± 0.04	17.31 ± 0.05	0.69 ± 0.00	446.89 ± 0.45
**Piñonera R-I**	1.35 ± 0.08	6.66 ± 0.27	0.78 ± 0.04	0.19 ± 0.02	0.48 ± 0.02	2.98 ± 0.21	206.03 ± 0.23	227.49 ± 4.51	1.03 ± 0.00	0.28 ± 0.03	1.34 ± 0.01	39.52 ± 0.59	37.09 ± 8.94	0.07 ± 0.00	3.78 ± 0.11	0.54 ± 0.07	689.33 ± 20.97	1.14 ± 0.46	4.54 ± 0.20	249.87 ± 0.65	271.24 ± 13.71	4.31 ± 0.18	2.37 ± 0.01	1.80 ± 0.06	695.01 ± 21.62
**Piñonera R-II**	1.55 ± 0.02	7.99 ± 0.01	0.78 ± 0.01	0.21 ± 0.01	0.48 ± 0.00	2.15 ± 0.11	206.89 ± 6.92	246.04 ± 7.66	1.37 ± 0.15	0.35 ± 0.01	1.58 ± 0.07	41.04 ± 0.40	30.98 ± 2.51	0.06 ± 0.00	4.42 ± 0.13	0.66 ± 0.01	746.34 ± 18.04	1.23 ± 0.05	3.90 ± 0.67	251.63 ± 7.19	285.01 ± 5.16	5.08 ± 0.14	2.96 ± 0.22	1.89 ± 0.03	751.46 ± 18.66
**Piñonera R-III**	3.60 ± 0.19	14.60 ± 0.45	0.54 ± 0.01	0.21 ± 0.02	0.49 ± 0.02	8.31 ± 0.54	259.81 ± 5.40	177.05 ± 2.62	1.48 ± 0.08	0.56 ± 0.04	1.49 ± 0.02	27.26 ± 3.17	19.49 ± 3.24	0.07 ± 0.00	5.50 ± 0.77	0.55 ± 0.15	827.80 ± 12.43	1.48 ± 0.34	9.10 ± 0.74	298.98 ± 9.30	211.13 ± 6.32	6.04 ± 0.92	2.97 ± 0.05	1.86 ± 0.01	838.38 ± 13.51
**Abou kanani R-I**	6.70 ± 0.29	2.96 ± 0.25	0.61 ± 0.09	0.29 ± 0.02	0.16 ± 0.21	5.50 ± 0.76	23.36 ± 4.18	11.79 ± 0.75	2.69 ± 0.13	0.09 ± 0.02	13.47 ± 0.22	70.68 ± 7.80	5.43 ± 0.33	0.19 ± 0.02	2.15 ± 0.06	1.03 ± 0.10	359.68 ± 22.65	0.51 ± 0.02	1.03 ± 0.98	106.24 ± 4.09	20.18 ± 0.83	3.18 ± 0.16	16.16 ± 0.34	1.34 ± 0.28	361.23 ± 21.68
**Abou kanani R-II**	3.95 ± 0.52	1.70 ± 0.15	0.24 ± 0.01	0.13 ± 0.02	0.29 ± 0.03	4.62 ± 0.11	22.47 ± 0.57	8.52 ± 1.06	2.46 ± 0.12	0.06 ± 0.01	10.91 ± 0.68	64.34 ± 4.65	2.66 ± 0.17	0.13 ± 0.00	5.16 ± 0.08	1.05 ± 0.00	213.87 ± 0.57	0.96 ± 0.10	2.01 ± 0.17	95.38 ± 5.85	12.88 ± 1.38	6.21 ± 0.07	13.37 ± 0.79	0.85 ± 0.07	216.85 ± 0.85
**Abou kanani R-III**	2.06 ± 0.07	0.76 ± 0.09	0.28 ± 0.01	0.17 ± 0.01	0.10 ± 0.02	4.61 ± 0.12	5.76 ± 1.47	2.53 ± 1.09	1.29 ± 0.08	0.03 ± 0.01	7.30 ± 0.24	18.14 ± 1.73	1.33 ± 2.55	0.06 ± 0.01	3.96 ± 0.17	0.63 ± 0.09	167.21 ± 12.41	0.97 ± 0.48	6.09 ± 0.55	30.57 ± 2.97	4.62 ± 3.20	4.59 ± 0.25	8.59 ± 0.30	0.64 ± 0.03	174.26 ± 12.48
**Fishomi R-I**	2.96 ± 0.28	5.67 ± 0.18	0.69 ± 0.01	0.23 ± 0.02	1.00 ± 0.08	3.03 ± 0.30	31.44 ± 4.15	26.57 ± 2.32	2.02 ± 0.01	0.10 ± 0.02	3.21 ± 0.07	48.59 ± 4.20	34.10 ± 0.94	0.27 ± 0.01	5.16 ± 0.17	0.88 ± 0.01	342.25 ± 2.16	4.30 ± 0.39	2.28 ± 0.81	86.02 ± 8.95	66.35 ± 3.44	6.03 ± 0.16	5.23 ± 0.08	2.29 ± 0.11	348.83 ± 2.58
**Fishomi R-II**	3.02 ± 0.06	5.11 ± 0.08	0.76 ± 0.01	0.21 ± 0.02	0.62 ± 0.01	2.14 ± 0.10	22.13 ± 1.30	21.27 ± 1.13	1.84 ± 0.00	0.10 ± 0.02	2.78 ± 0.01	52.56 ± 2.23	38.36 ± 1.24	0.19 ± 0.02	6.38 ± 0.23	1.05 ± 0.06	339.37 ± 31.99	3.17 ± 0.66	1.45 ± 0.01	79.86 ± 3.37	64.74 ± 0.19	7.43 ± 0.28	4.62 ± 0.00	1.88 ± 0.01	343.99 ± 31.34
**Fishomi R-III**	3.19 ± 0.06	4.26 ± 0.12	0.37 ± 0.02	0.17 ± 0.02	0.27 ± 0.01	1.40 ± 0.01	18.69 ± 1.03	15.17 ± 0.99	1.39 ± 0.09	0.10 ± 0.02	3.08 ± 0.07	115.09 ± 3.56	49.34 ± 4.04	0.10 ± 0.00	7.79 ± 0.45	0.87 ± 0.13	288.41 ± 5.79	3.12 ± 0.62	0.54 ± 0.12	138.36 ± 4.65	68.77 ± 5.14	8.65 ± 0.58	4.47 ± 0.16	1.01 ± 0.02	292.07 ± 5.05
**Picual R-I**	1.41 ± 0.03	1.24 ± 0.14	0.47 ± 0.02	0.10 ± 0.00	1.11 ± 0.01	2.48 ± 0.07	43.63 ± 1.16	27.35 ± 0.80	0.81 ± 0.08	0.03 ± 0.01	2.40 ± 0.53	158.95 ± 0.52	94.35 ± 8.38	0.12 ± 0.01	4.27 ± 0.03	1.33 ± 0.04	237.29 ± 20.50	3.03 ± 0.67	6.28 ± 0.72	206.46 ± 1.65	122.94 ± 7.73	5.60 ± 0.06	3.21 ± 0.61	1.83 ± 0.02	246.61 ± 20.45
**Picual R-II**	1.24 ± 0.07	2.32 ± 0.17	0.35 ± 0.01	0.09 ± 0.00	1.25 ± 0.02	2.24 ± 0.16	18.99 ± 0.03	16.18 ± 0.15	0.88 ± 0.12	0.01 ± 0.00	2.21 ± 0.46	107.58 ± 0.17	77.48 ± 3.18	0.26 ± 0.00	5.56 ± 0.03	1.47 ± 0.08	206.22 ± 6.61	2.98 ± 0.51	12.79 ± 0.85	130.05 ± 0.42	95.98 ± 3.19	7.04 ± 0.05	3.10 ± 0.59	1.96 ± 0.02	221.98 ± 5.25
**Picual R-III**	2.19 ± 0.04	4.64 ± 0.01	0.35 ± 0.02	0.06 ± 0.00	0.92 ± 0.01	2.04 ± 0.03	16.26 ± 0.05	11.84 ± 0.22	0.70 ± 0.12	0.01 ± 0.00	2.81 ± 0.31	102.20 ± 0.37	62.32 ± 4.71	0.28 ± 0.01	6.41 ± 0.01	1.13 ± 0.02	230.16 ± 9.80	2.88 ± 0.36	13.18 ± 1.35	122.70 ± 0.43	78.80 ± 4.49	7.53 ± 0.02	3.51 ± 0.42	1.62 ± 0.04	246.22 ± 10.80
**Arbequina R-I**	1.16 ± 0.01	1.51 ± 0.04	0.96 ± 0.01	0.20 ± 0.00	0.62 ± 0.04	10.49 ± 0.06	131.42 ± 3.57	82.60 ± 2.20	2.67 ± 0.08	0.09 ± 0.00	28.05 ± 0.01	17.32 ± 1.53	1.57 ± 0.04	0.11 ± 0.00	7.20 ± 0.08	2.38 ± 0.10	288.08 ± 26.24	0.08 ± 0.12	1.69 ± 0.53	160.39 ± 1.99	85.68 ± 2.12	9.59 ± 0.18	30.72 ± 0.07	1.98 ± 0.02	289.86 ± 26.13
**Arbequina R-II**	1.81 ± 0.04	1.45 ± 0.08	0.70 ± 0.03	0.15 ± 0.00	0.50 ± 0.01	22.01 ± 0.33	98.74 ± 1.70	54.18 ± 1.53	2.40 ± 0.15	0.12 ± 0.01	24.63 ± 0.61	12.06 ± 2.09	0.86 ± 0.32	0.14 ± 0.01	9.96 ± 0.54	2.72 ± 0.29	246.81 ± 0.68	0.86 ± 0.85	0.73 ± 0.61	134.62 ± 4.17	56.49 ± 1.93	12.68 ± 0.83	27.03 ± 0.76	1.61 ± 0.06	248.41 ± 2.14
**Arbequina R-III**	0.58 ± 0.01	1.13 ± 0.04	0.49 ± 0.00	0.11 ± 0.00	0.33 ± 0.00	11.92 ± 0.01	26.48 ± 0.22	25.23 ± 0.50	2.18 ± 0.07	0.03 ± 0.00	19.36 ± 0.07	5.01 ± 0.92	0.68 ± 0.10	0.14 ± 0.00	9.57 ± 0.01	2.29 ± 0.12	223.48 ± 7.88	0.13 ± 0.19	0.34 ± 0.49	43.99 ± 1.12	27.04 ± 0.56	11.87 ± 0.12	21.54 ± 0.00	1.09 ± 0.01	223.96 ± 8.56
**UCI-20 R-I**	8.83 ± 0.11	9.30 ± 2.18	0.34 ± 0.00	0.09 ± 0.00	1.18 ± 0.03	3.25 ± 0.55	20.35 ± 6.41	24.70 ± 7.90	4.87 ± 0.10	0.02 ± 0.00	12.69 ± 0.63	68.81 ± 1.94	53.15 ± 5.57	0.12 ± 0.01	7.81 ± 0.70	2.40 ± 0.18	307.77 ± 0.80	4.26 ± 0.58	2.37 ± 0.66	101.24 ± 7.91	87.14 ± 11.29	10.21 ± 0.88	17.56 ± 0.53	1.75 ± 0.01	314.41 ± 0.44
**UCI-20 R-II**	4.44 ± 0.70	5.13 ± 0.71	0.31 ± 0.03	0.06 ± 0.01	0.74 ± 0.07	2.87 ± 0.17	13.13 ± 2.57	12.14 ± 2.08	4.31 ± 0.29	0.01 ± 0.00	12.75 ± 1.12	41.91 ± 5.48	28.97 ± 3.61	0.22 ± 0.03	8.42 ± 0.91	1.66 ± 0.17	232.49 ± 21.84	1.34 ± 0.37	2.74 ± 2.82	62.36 ± 8.92	46.24 ± 6.40	10.08 ± 1.08	17.06 ± 1.41	1.33 ± 0.13	236.58 ± 24.28
**UCI-20 R-III**	2.67 ± 0.30	5.13 ± 0.05	0.40 ± 0.01	0.05 ± 0.00	0.37 ± 0.01	2.70 ± 0.14	2.67 ± 0.30	2.73 ± 0.05	3.42 ± 0.08	0.03 ± 0.01	10.55 ± 0.31	20.48 ± 0.01	11.11 ± 1.17	0.09 ± 0.00	14.24 ± 0.09	2.08 ± 0.04	217.22 ± 10.35	2.01 ± 0.05	0.91 ± 0.12	28.52 ± 0.73	18.97 ± 1.27	16.32 ± 0.05	13.97 ± 0.39	0.94 ± 0.03	220.14 ± 10.29
**UCI-21 R-I**	10.45 ± 1.19	3.98 ± 0.16	0.84 ± 0.02	0.14 ± 0.01	0.30 ± 0.00	2.13 ± 0.22	47.65 ± 0.20	24.57 ± 0.62	1.45 ± 0.09	0.02 ± 0.01	4.47 ± 0.50	125.00 ± 1.15	42.33 ± 1.39	0.03 ± 0.00	8.75 ± 0.15	4.82 ± 0.47	338.79 ± 41.91	2.20 ± 0.33	2.24 ± 1.15	185.23 ± 0.06	70.88 ± 0.61	13.57 ± 0.31	5.91 ± 0.58	1.33 ± 0.01	343.24 ± 41.09
**UCI-21 R-II**	6.00 ± 0.81	3.07 ± 0.34	0.94 ± 0.03	0.11 ± 0.00	0.24 ± 0.00	2.92 ± 0.23	47.70 ± 5.29	17.84 ± 1.51	1.54 ± 0.07	0.02 ± 0.01	4.44 ± 0.24	81.35 ± 4.32	23.22 ± 2.63	0.07 ± 0.00	9.28 ± 0.11	4.06 ± 0.15	258.75 ± 1.17	5.47 ± 1.20	1.07 ± 0.63	137.97 ± 8.58	44.12 ± 3.80	13.34 ± 0.04	5.98 ± 0.31	1.39 ± 0.04	265.29 ± 0.66
**UCI-21 R-III**	3.19 ± 0.69	4.07 ± 0.14	1.08 ± 0.02	0.09 ± 0.01	0.25 ± 0.01	4.00 ± 0.35	20.98 ± 0.46	6.15 ± 0.11	1.45 ± 0.10	0.02 ± 0.00	3.89 ± 0.02	37.86 ± 0.38	11.09 ± 3.00	0.13 ± 0.00	10.33 ± 0.46	3.51 ± 0.35	192.03 ± 12.92	5.80 ± 0.21	0.78 ± 0.32	66.02 ± 0.19	21.31 ± 3.04	13.84 ± 0.81	5.34 ± 0.12	1.57 ± 0.03	198.60 ± 12.39
**UCI-42 R-I**	9.70 ± 0.50	10.12 ± 0.34	0.28 ± 0.02	0.15 ± 0.01	0.42 ± 0.00	1.76 ± 0.16	42.62 ± 2.01	62.39 ± 1.57	0.64 ± 0.20	0.05 ± 0.02	4.12 ± 0.12	200.53 ± 2.04	213.27 ± 5.55	0.03 ± 0.00	2.64 ± 0.01	0.83 ± 0.00	347.41 ± 7.38	0.78 ± 0.54	0.27 ± 0.38	254.62 ± 0.63	285.79 ± 3.65	3.48 ± 0.01	4.76 ± 0.08	0.93 ± 0.05	348.46 ± 7.54
**UCI-42 R-II**	12.89 ± 0.65	9.18 ± 0.53	0.16 ± 0.01	0.08 ± 0.00	0.40 ± 0.01	1.81 ± 0.08	37.49 ± 0.52	41.14 ± 0.06	0.52 ± 0.18	0.06 ± 0.02	2.56 ± 0.35	297.17 ± 5.16	220.81 ± 6.98	0.02 ± 0.00	3.53 ± 0.34	0.92 ± 0.08	344.38 ± 4.90	3.25 ± 0.52	1.05 ± 0.50	349.36 ± 5.12	271.13 ± 6.51	4.45 ± 0.43	3.08 ± 0.53	0.72 ± 0.04	348.69 ± 4.897
**UCI-42 R-III**	8.37 ± 0.79	3.34 ± 0.02	0.14 ± 0.00	0.05 ± 0.00	0.79 ± 0.02	2.55 ± 0.01	29.93 ± 1.19	16.76 ± 0.79	0.67 ± 0.04	0.08 ± 0.01	6.83 ± 0.08	212.01 ± 8.51	66.62 ± 5.01	0.08 ± 0.01	8.72 ± 0.06	0.99 ± 0.04	225.77 ± 2.50	1.62 ± 0.28	0.31 ± 0.36	252.86 ± 10.49	86.72 ± 4.24	9.71 ± 0.09	7.50 ± 0.12	1.15 ± 0.02	227.70 ± 2.417
